# Cofactor‐Assisted Artificial Enzyme with Multiple Li‐Bond Networks for Sustainable Polysulfide Conversion in Lithium–Sulfur Batteries

**DOI:** 10.1002/advs.202104205

**Published:** 2021-11-07

**Authors:** Suya Zhou, Shuo Yang, Dong Cai, Ce Liang, Shuang Yu, Yue Hu, Huagui Nie, Zhi Yang

**Affiliations:** ^1^ Key Laboratory of Carbon Materials of Zhejiang Province Wenzhou University Wenzhou 325035 China; ^2^ College of Electrical and Electronic Engineering Wenzhou University Wenzhou 325035 China

**Keywords:** biomimetic catalysts, covalent amide bonds, FeN_5_ coordination structures, lithium–sulfur batteries, multiple Li‐bond networks

## Abstract

Lithium–sulfur batteries possess high theoretical energy density but suffer from rapid capacity fade due to the shuttling and sluggish conversion of polysulfides. Aiming at these problems, a biomimetic design of cofactor‐assisted artificial enzyme catalyst, melamine (MM) crosslinked hemin on carboxylated carbon nanotubes (CNTs) (i.e., [CNTs–MM–hemin]), is presented to efficiently convert polysulfides. The MM cofactors bind with the hemin artificial enzymes and CNT conductive substrates through FeN_5_ coordination and/or covalent amide bonds to provide high and durable catalytic activity for polysulfide conversions, while *π*–*π* conjugations between hemin and CNTs and multiple Li‐bond networks offered by MM endow the cathode with good electronic/Li^+^ transmission ability. This synergistic mechanism enables rapid sulfur reaction kinetics, alleviated polysulfide shuttling, and an ultralow (<1.3%) loss of hemin active sites in electrolyte, which is ≈60 times lower than those of noncovalent crosslinked samples. As a result, the Li–S battery using [CNTs–MM–hemin] cathode retains a capacity of 571 mAh g^−1^ after 900 cycles at 1C with an ultralow capacity decay rate of 0.046% per cycle. Even under raising sulfur loadings up to 7.5 mg cm^−2^, the cathode still can steadily run 110 cycles with a capacity retention of 83%.

## Graphical abstract

A cofactor‐assisted artificial enzyme with multiple Li‐bond networks, melamine (MM) crosslinked hemin on carboxylated carbon nanotubes (CNTs) ([CNTs–MM–hemin]), as a catalyst is introduced into lithium–sulfur (Li–S) batteries. The [CNTs–MM–hemin]‐based cathode significantly accelerates the sulfur redox reaction kinetics and suppresses the polysulfide shuttle, thereby improving the electrochemical performance of Li–S batteries remarkably, even under high sulfur loadings.

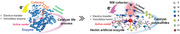

## 1 Introduction

The electrochemical redox reaction of sulfur in lithium–sulfur (Li–S) batteries undergoes a complicated multiphase evolution and a multistep electron‐transfer process, which accommodates more Li^+^ than current transition metal‐based cathodes of Li‐ion batteries.^[^ ^1^ ^]^ It has been commonly accepted that, during the first discharge, solid elemental sulfur (S_8_) on a cathode accepts electrons, generating soluble long‐chain lithium polysulfides (LiPSs) through cleavage of the sulfur ring, and then the long‐chain LiPSs are further reduced to short‐chain LiPSs and finally to insoluble Li_2_S_2_/Li_2_S products at lower potentials; when the battery is charged, the Li_2_S_2_/Li_2_S is reversibly oxidized to various LiPSs, and then back to S_8_.^[^ ^2^, ^3^ ^]^ This sulfur redox reaction (SRR) is essential for Li–S batteries with an ultrahigh theoretical capacity (1675 mAh g^−1^).^[^ ^4^, ^5^ ^]^ At present, the greatest challenges to gain high capacity and long life of practical Li–S systems are the sluggish sulfur redox kinetics and the shuttle effect of the intermediate LiPSs.^[^ ^6^, ^7^ ^]^ Although some polar materials, such as transition metal oxides,^[^ ^8^, ^9^ ^]^ metal organic frameworks,^[^ ^10^, ^11^, ^12^ ^]^ and polymers,^[^ ^13^, ^14^ ^]^ which can supply a large amount of catalytic and adsorption active sites for LiPSs, have been developed as cathode catalysts to accelerate the LiPS conversion and mitigate the shuttle effect in a certain degree, the enhanced electrochemical performances of Li–S batteries with these catalysts are still very limited. Therefore, designing highly active and durable cathode catalysts to propel the complex SRR chemistry is the necessary route to realize the full theoretical capacity and long cycle life of Li–S batteries.

Enzyme has evolved in complex natural environments over three billion years, and is therefore an effective catalyst to be used for promoting fundamental processes of life.^[^ ^15^ ^]^ Rising demands for efficient electrocatalysis call for new enzymatic functions that may not be relevant in the biological world. To expand the biocatalytic space to electrocatalytic chemistry, scientists have started to structurally and functionally mimic the natural enzyme by human‐invented chemistry strategy to construct artificial enzymes.^[^ ^16^ ^]^ Many of the reported artificial enzymes, which are designed via either construction of different catalytic active centers or directed evolution engineering, have been demonstrated to catalyze electrochemical oxygen reduction reactions,^[^ ^17^ ^]^ CO_2_ conversions, and the reduction of N_2_ to NH_3_.^[^ ^18^ ^]^ However, the high catalytic activity and high stability of the artificial enzymes are usually difficult to balance, the inactivation and loss problems of the active centers in the artificial enzymes remain a challenge. Recently, with the in‐depth research on natural enzymes, it has been found that cofactors, as indispensable participants in enzyme‐catalyzed reactions, play an important role in enhancing the catalytic functions and stability of the natural enzymes, especially in catalyzing the diverse, challenging chemical transformations in water environment of life systems,^[^ ^19^ ^]^ owing to their covalent immobilization of enzyme and strong electronic/ionic transmission through hydrogen‐bond networks. The good immobilization capability toward catalytic active sites and perfect electronic/ionic transport are highly desired for an ideal Li–S reaction, in particular, for the struggling conversion reaction of short‐chain LiPSs, in which a large amount of Li^+^ need to be supplemented.^[^ ^2^ ^]^ Consequently, it is speculated that the design of hydrogen‐bond network‐like multiple Li‐bond networks and introduce the networks into the Li–S chemistry by a covalent crosslinking method may be a good strategy for realizing efficient and steady LiPS conversion and alleviating the shuttle effect in Li–S batteries.

Inspired by the cofactor‐assisted enzyme, after combining with the advantages of N‐rich melamine (MM) molecules in forming multiple Li‐bond networks, we propose a new biomimetic catalyst for highly efficient LiPS conversion, which is realized by crosslinking lithiophilic MM cofactor with the hemin artificial enzyme modified carboxylated carbon nanotubes (CNTs) via an amidation reaction, denoted as [CNTs–MM–hemin]. A series of characterizations, in situ spectroscopic studies, and theoretical calculations reveal: 1) A successful formation of the amide bonds between MM, hemin, and carboxylated CNTs helps to establish a period and covalently crosslinked network structure, inhibiting the dissolution of hemin artificial enzyme in ether‐based solvents and thus maintaining the relatively high and stable catalytic activities for LiPS conversions; 2) a FeN_5_ configuration formed by Fe–N coordination between hemin and MM significantly enhances the conversion of long‐chain LiPSs to short‐chain LiPSs; 3) a large number of different Li‐bonds are formed between LiPSs and hemin and/or MM, constructing multiple Li‐bond networks, which is beneficial for accelerating electron transfer/Li^+^ supplementation in the system and boosting the conversion of short‐chain LiPSs to insoluble Li_2_S_2_/Li_2_S. As a result of this synergistic effect, the [CNTs–MM–hemin]‐based Li–S batteries exhibit excellent electrochemical performance in terms of cycling stability. While Li‐bonds in Li–S chemistry have attracted great attentions, to the best knowledge, the concept of multiple Li‐bond networks and the corresponding study are sorely lacked in the rechargeable battery community. This work will pave the way for designing cofactor‐assisted artificial enzyme catalysts with multiple Li‐bond networks for boosting the electrochemical performance of practical Li–S batteries.

## 2 Results and Discussion

### 2.1 Synthesis and Characterization of [CNTs–MM–Hemin] Composite

**Figure 1:**
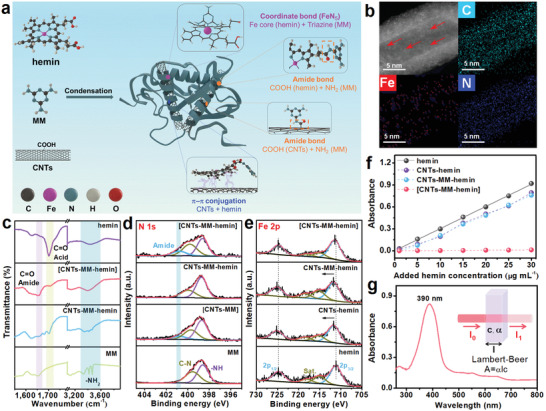
Schematic illustration and structural and stability characterization of [CNTs–MM–hemin] composites. a) Schematic of the synthetic route for [CNTs–MM–hemin] composite and the amplification of physical interaction, covalent, and coordinated sites of the resulting composite. b) HAADF‐STEM image and the corresponding EDX elemental mapping images of C, Fe, and N for [CNTs–MM–hemin]. c) FTIR spectra of hemin, [CNTs–MM–hemin], CNTs–MM–hemin, and MM. d) N 1s XPS spectra of [CNTs–MM–hemin], CNTs–MM–hemin, [CNTs–MM], and MM. e) Fe 2p XPS spectra of [CNTs–MM–hemin], CNTs–MM–hemin, CNTs–hemin, and hemin. f) Dependence of the hemin addition on the UV–vis absorbance peak at different composites in a binary solvent of DOL and DME (v/v = 1:1). g) UV–vis absorption spectrum of hemin dissolved in the DOL/DME solvent.

As schematically illustrated in **Figure** **1**a, [CNTs–MM–hemin] composite was synthesized by a simple condensation method (the detailed synthetic process can be found in the Experimental Section). For comparison, the sample without hemin (denoted as [CNTs–MM]) and the samples without condensation method (denoted as CNTs–MM–hemin and CNTs–hemin, in which hemin is physically bonded to its host) were also prepared. In high‐angle annular dark‐field scanning transmission electron microscopy (HAADF‐STEM) images of the obtained composite (Figure 1b; Figure S1, Supporting Information), the marked brighter points could be corresponded to the well‐dispersed Fe. The homogeneous spatial distributions of Fe, N, and C atoms are further evidenced by the energy dispersive X‐ray spectroscopy (EDX) elemental mappings (Figure 1b; Figure S2, Supporting Information), indicating hemin molecules are distributed evenly throughout CNT surfaces.

The [CNTs–MM–hemin] composite was further examined by Fourier transform infrared (FTIR) and X‐ray photoelectron spectroscopy (XPS) to confirm the association of MM to hemin and carboxylated CNTs. In Figure 1c, the strong peaks (at 1704 cm^−1^) assigned to the carboxylic stretch of carboxylic group of hemin (C═O (acid))^[^ ^20^ ^]^ are observed in hemin and CNTs–MM–hemin, and the sharp peaks (at 3300–3500 cm^−1^) ascribed to the amino groups of MM can be clearly found in MM and CNTs–MM–hemin. Whereas, the peaks at 1704 and 3300–3500 cm^−1^ in [CNTs–MM–hemin] are significantly weakened, while the C═O (amide) peak^[^ ^20^ ^]^ at ≈1656 cm^−1^ is enhanced. These findings suggest a successful formation of the covalent amide bond when the amino group of MM reacts with the carboxylic group of the hemin and carboxylated CNTs via condensation reaction, which is also confirmed by the amide peak at ≈400.8 eV in the N 1s XPS spectra of [CNTs–MM–hemin] and [CNTs–MM] (Figure 1d). In Figure 1e, compared with hemin, the Fe 2p peaks of CNTs–hemin and CNTs–MM–hemin significantly shift to higher binding energy, indicating that the physical *π*–*π* conjugation interaction between CNTs and hemin/MM successfully occurs.^[^ ^21^ ^]^ For [CNTs–MM–hemin], it is found that its Fe 2p peaks significantly shift to lower binding energy compared with those of CNTs–MM–hemin (Figure 1e), and its Cl 2p peaks disappear (Figure S3, Supporting Information), combined with the theoretical calculation of dechlorination reaction energy (Figure S4, Supporting Information), which suggests that the weak Fe—Cl bond in hemin is easily cleaved during condensation reaction and the N‐rich MM facilitates the coordination with Fe atom of hemin through N_1_ site, probably forming an unique FeN_5_ site.^[^ ^21^ ^]^

To explore the immobilization effect of the multiple connections, including covalent amide bond, physical *π*–*π* conjugation, and Fe—N coordination bond, on the highly soluble hemin in ether‐based electrolyte, UV–vis absorption spectroscopic studies were carried out on hemin, CNTs–hemin, CNTs–MM–hemin, and [CNTs–MM–hemin] in a binary solvent of 1,3‐dioxolane (DOL) and dimethyl ether (DME) (DOL/DME, v/v = 1:1). Figure 1f and Figure S5 in the Supporting Information give the intensity variation in soret band^[^ ^22^ ^]^ for hemin (≈390 nm, as shown in Figure 1g) with the added hemin concentration (1–30 µg mL^−1^). Clearly, [CNTs–MM–hemin] exhibits an extremely low solubility of hemin (<1.3%) among the three composites, which is around 60 times lower than those of noncovalent crosslinked samples (76% for CNTs–MM–hemin, and 79.5% for CNTs–hemin), manifesting [CNTs–MM–hemin] has a robust ability to immobilize hemin and inhibit its dissolution in ether‐based solvents.

On the basis of the above spectral results, it is reasonable to conclude that in [CNTs–MM–hemin], MM acts as a cofactor to grab hemin artificial enzyme and carboxylated CNTs with covalent amide bonds, a FeN_5_ configuration is formed by Fe–N coordination between hemin and MM, and the physical *π*–*π* interactions between CNTs and the molecules (hemin and MM) also exist to accelerate electron transfer in the system, as presented in Figure 1a. With the aid of the multiple (covalent, coordinated, physical) connections, [CNTs–MM–hemin] with a periodic covalent network structure can efficiently inhibit the dissolution of hemin in ether‐based solvents.

### 2.2 Interaction between Polysulfides and [CNTs–MM–Hemin]

**Figure 2:**
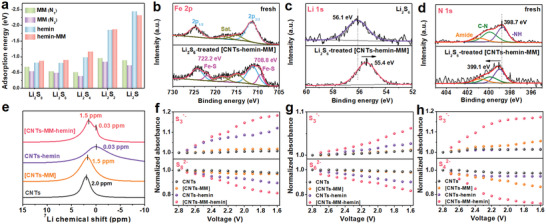
Interaction and catalytic ability of the catalysts toward LiPSs. a) The adsorption energies (*E*_ads_) between Li_2_S*_n_* (*n* = 8, 6, 4, 2, 1) and hemin–MM, hemin, and MM with N_1_ and N_2_ sites. b) Fe 2p XPS spectra of the fresh and Li_2_S_6_‐treated [CNTs–MM–hemin]. c) Li 1s XPS spectra of the Li_2_S_6_ and Li_2_S_6_‐treated [CNTs–MM–hemin]. d) N 1s XPS spectra of the fresh and Li_2_S_6_‐treated [CNTs–MM–hemin]. e) ^7^Li solid‐state NMR spectra of Li_2_S_6_‐treated CNTs, [CNTs–MM], CNTs–hemin, and [CNTs–MM–hemin], which were immersed into the Li_2_S_6_ solution for 24 h and then evaporated in an Ar‐filled glovebox for dryness. The normalized absorbance of f) S_8_^2−^ (492 nm) and S_3_^*−^ (617 nm) in Li_2_S_8_ solution, g) S_6_^2−^ (475 nm) and S_3_^*−^ (617 nm) in Li_2_S_6_ solution, and h) S_4_^2−^ (420 nm) and S_3_^*−^ (617 nm) in Li_2_S_4_ solution on CNT, [CNTs–MM], CNTs–hemin, and [CNTs–MM–hemin] electrodes during discharge.

Encouraged by the exciting immobilization capability of [CNTs–MM–hemin] toward catalytic active sites of hemin, its adsorption and catalytic effects on LiPSs were evaluated experimentally and theoretically. Theoretical calculations based on density functional theory (DFT) were performed to model the adsorption of Li_2_S*_n_* molecules (*n* = 8, 6, 4, 2, 1) on the surfaces of hemin–MM, hemin, and MM with N_1_ and N_2_ site configurations, respectively. The most favorable optimized local atomic structure and conformation of adsorbed Li_2_S*_n_* (*n* = 8, 6, 4, 2, 1) on the four surfaces are shown in Figure S6 in the Supporting Information. It is found that MM binds with hemin via a Fe—N_1_ bond, and the Li_2_S*_n_* molecule (*n* = 8, 6, 4, 2, 1) adsorbs on hemin–MM, hemin, or MM through Fe—S and Li—N bonds. From **Figure** **2**a and Figure S6 in the Supporting Information, obviously, Li_2_S*_n_* molecules (*n* = 8, 6, 4, 2, 1) adsorb onto the hemin–MM surface with the adsorption energy (*E*_ads_) of −0.87 to −2.32 eV, which are generally lower than those of hemin (−0.82 to −2.45 eV) and MM (−0.68 to −0.89 eV), indicating that hemin–MM theoretically has the strongest chemical entrapment ability toward sulfur species mainly due to the Fe—S and Li—N bonds, and should have a positive effect for suppressing the shuttle effect. Further, the LiPS chemisorption capabilities of CNTs, [CNTs–MM], and [CNTs–MM–hemin] composites were experimentally characterized by soaking the three composites into Li_2_S_6_ solution (Figure S7, Supporting Information). A decolorized Li_2_S_6_ solution is observed in the presence of [CNTs–MM–hemin], while the solution with [CNTs–MM] still demonstrates light yellow color, and that with CNTs keeps dark yellow in color. Such a remarkable contrast indicates that [CNTs–MM–hemin] catalyst has the best adsorbability for LiPSs, completely consistent with the above DFT calculations (Figure 2a; Figure S6, Supporting Information). To further analyze the intrinsic chemical interaction of LiPSs with [CNTs–MM–hemin], ex situ XPS study was executed after Li_2_S_6_ adsorption for 24 h. The S 2p peak of Li_2_S_6_‐treated [CNTs–MM–hemin] at 167.2 eV (Figure S8, Supporting Information) is in accord with the binding energy of thiosulfate, suggesting an effective polysulfide conversion on the [CNTs–MM–hemin] surface.^[^ ^23^ ^]^ The Fe 2p peaks for [CNTs–MM–hemin] display significant changes after Li_2_S_6_ treatment (Figure 2b). Two new peaks at 708.8 and 722.2 eV could be ascribed to Fe—S bond,^[^ ^24^ ^]^ which results from the interaction between the Fe atom of hemin and the S atom of LiPSs. The Li 1s peak of Li_2_S_6_‐treated [CNTs–MM–hemin] is shifted toward lower binding energy by 0.7 eV compared with that of the pure Li_2_S_6_ (56.1 eV; Figure 2c), as expected, its N 1s peak is shifted toward higher binding energy (Figure 2d), implying a strong electron transfer from the N atoms of hemin and/or MM to Li^+^ or LiPSs that results in Li‐bond formation, namely the formation of the Li···N bond, according to previous reports.^[^ ^25^ ^]^ A ^7^Li NMR investigation of the different Li···N bonds was further conducted since different types of N environment exist in hemin and MM. The narrow signal due to the soluble Li^+^ appears at ≈2.0 ppm in the spectrum of the Li_2_S_6_ solution (Figure S9, Supporting Information). In the case of Li_2_S_6_‐treated CNTs, no chemical shift is observed expect for a peak broadening, while the downshifts of the ^7^Li resonance in different degrees for Li_2_S_6_‐treated [CNTs–MM] and Li_2_S_6_‐treated CNTs–hemin in Figure 2e indicate that the Li nucleus is more shielded by different N environments (forming different Li···N bonds) in MM and hemin.^[^ ^26^ ^]^ In case of Li_2_S_6_‐treated [CNTs–MM–hemin], both ^7^Li signals of [CNTs–MM] and CNTs–hemin remain, demonstrating the formation of multiple Li···N bonds between the [CNTs–MM–hemin] and LiPSs. According to the principle of NMR,^[^ ^27^ ^]^ the solid Li_2_S components generally result in broader signals in NMR spectra due to the presence of anisotropic interactions. However, the interactions are averaged out on the NMR time scale by fast tumbling of the soluble polysulfide species, generally resulting in narrow line widths for the solution components. Thus, the narrowing in the ^7^Li peak at ≈1.5 ppm could be attributed to the higher Li^+^ mobility in [CNTs–MM–hemin]. The above findings demonstrate that [CNTs–MM–hemin] can effectively adsorb LiPSs through the Fe—S and multiple Li···N bonds, not only avoiding the LiPS shuttling but also ensuring a faster Li^+^ migration/supplement in the system.

The catalytic effect of the [CNTs–MM–hemin] on the LiPS redox kinetics was also revealed by the cycle voltammogram (CV) profiles in the Li_2_S_6_ symmetric cells (Figure S10, Supporting Information). All the Li_2_S_6_ symmetric cells show more obvious current response in contrast to the CNT symmetric cell without Li_2_S_6_ electrolyte. Particularly, the electrodes with hemin, including CNTs–hemin, CNTs–MM–hemin, and [CNTs–MM–hemin], exhibit higher redox currents than those of [CNTs–MM] and CNTs, meanwhile, the redox currents between CNTs–MM–hemin and CNTs–hemin (or between [CNT‐MM] and CNT) are almost comparable, implying superiority of hemin, but not MM, in catalyzing LiPS redox kinetics. Impressively, the [CNTs–MM–hemin] electrode demonstrates the largest redox current, verifying that the special FeN_5_ configuration in [CNTs–MM–hemin] significantly enhances the LiPS conversion reaction kinetics. This point can be further confirmed by the in situ UV–vis absorption spectroscopic results. Figures S11–S13 in the Supporting Information present the in situ UV–vis absorption spectra of various dissolved LiPS species (Li_2_S*_n_*, *n* = 8, 6, 4) at the four electrodes, respectively, while Figure 2f–h displays the normalized intensities of the absorbance peaks at 492, 475, 420, and 617 nm, corresponding to the S_8_^2−^, S_6_^2−^, S_4_^2−^, and S_3_^*−^ ions,^[^ ^28^ ^]^ respectively, as a function of potential. According to the drastically decreased intensities of S_8_^2−^ and S_6_^2−^ and increased intensity of S_3_^*−^ on the electrodes containing hemin (CNTs–hemin and [CNTs–MM–hemin]) and the comparable intensities of S_8_^2−^, S_6_^2−^, and S_3_^*−^ between [CNTs–MM] and CNTs during discharge (Figure 2f,g), it is deduced that compared to MM, hemin has better catalytic capability to covert long‐chain LiPSs (S_8_^2−^ and S_6_^2−^) to S_3_^*−^ radicals, especially when a FeN_5_ configuration is formed in [CNTs–MM–hemin]. For the conversion of short‐chain LiPSs (S_4_^2−^), although both hemin and MM show very limited catalytic effect (as found in purple and orange dots in Figure 2h), after the condensation reaction, [CNTs–MM–hemin] also manifests an amazing catalytic potential in converting short‐chain S_4_^2−^ ions to S_3_^*−^ radicals (as shown with the pink dots in Figure 2h).

### 2.3 Electrochemical Performance

**Figure 3:**
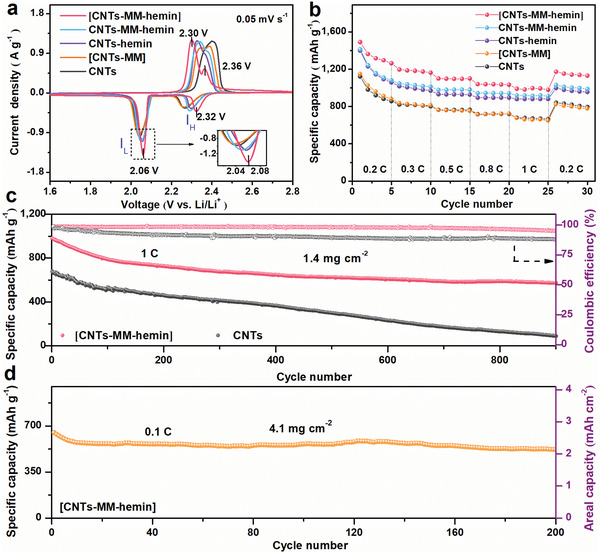
Electrochemical performances of Li–S batteries with different catalysts. a) The fourth cycle of CVs for the cathodes containing [CNTs–MM–hemin], CNTs–MM–hemin, CNTs–hemin, [CNTs–MM], and CNTs, respectively. Right inset is higher magnification of the reduction current between 2.01 and 2.09 V. b) The rate capabilities of the five cathodes. c) The long‐term cycling performance of the cathode containing [CNTs–MM–hemin] and CNTs over 900 cycles at 1C. d) Cycling stability of the cathode containing [CNTs–MM–hemin] with a sulfur mass loading of 4.1 mg cm^−2^ measured at a rate of 0.1C for 200 cycles.

To verify the beneficial effect of the [CNTs–MM–hemin] catalyst on improving the performance of Li–S batteries, CVs, galvanostatic charge/discharge voltage profiles, and rate performance of the batteries using [CNTs–MM–hemin], CNTs–MM–hemin, CNTs–hemin, [CNTs–MM], and CNTs modified cathodes are compared in **Figure** **3** and Figures S14 and S15 in the Supporting Information. The reduction/oxidation peaks for the cathodes containing hemin (CNTs–hemin, CNTs–MM–hemin, and [CNTs–MM–hemin]) are more positive/negative than those of [CNTs–MM] and CNTs modified cathodes (Figure 3a), indicating that hemin artificial enzyme as a catalyst may efficiently facilitate the catalytic conversion between sulfur and Li_2_S_2_/Li_2_S, in agreement with our previous reports.^[^ ^21^, ^29^, ^30^ ^]^ The CV plot of the battery with [CNTs–MM–hemin] cathode presents the sharpest and most positive/negative reduction/oxidation peaks, the smallest voltage hysteresis (Δ*V*) and the highest collection coefficient (*I*_L_/*I*_H_) compared to the batteries with the other four cathodes (Figure 3a; Table S1, Supporting Information), suggesting the [CNTs–MM–hemin] cathode greatly enhances the sulfur redox reaction kinetics within the battery.^[^ ^31^ ^]^ All charge/discharge profiles of the five batteries at 0.2C exhibit two distinct discharge and one charge plateaus (Figure S15a, Supporting Information), corresponding to the typical multistep conversion reactions of sulfur, in consistent well with the above CV results (Figure 3a; Figure S14, Supporting Information). Obviously, the charge/discharge curves of the battery with [CNTs–MM–hemin] cathode reveal longest plateaus and lowest polarization voltage (Δ*E*; Figure S15b, Supporting Information), reflecting the capacity improvement and faster reaction kinetics contributed by the catalysis of [CNTs–MM–hemin] composites. In Figure 3b, the discharge capacities of [CNTs–MM–hemin] cathode are 1490, 1196, 1100, 1038, and 982 mAh g^−1^ at 0.2C, 0.3C, 0.5C, 0.8C, and 1C (1C = 1675 mAh g^−1^), respectively. As the current density is restored to 0.2C, a specific capacity of 1172 mAh g^−1^ is recovered, which is substantially higher than those of the CNTs–MM–hemin, CNTs–hemin, [CNTs–MM] and CNT cathodes. The ultrahigh reversible capacity of the [CNTs–MM–hemin] cathode strongly indicates the enhanced sulfur utilization and accelerated redox kinetics of LiPSs at [CNTs–MM–hemin] cathode surface. For the long‐term cycling performance, the [CNTs–MM–hemin], CNTs–MM–hemin, and CNT cathodes with sulfur loading of ≈1.4 mg cm^−2^ were further evaluated at 1C (Figure 3c; Figure S16, Supporting Information). The [CNTs–MM–hemin] cathode shows the best cycling stability among them. It delivers the initial and retained discharge capacities of 978 and 571 mAh g^−1^ after 900 cycles, corresponding to a capacity decay ratio of 0.046% per cycle. To the best of our knowledge, such a good cycling stability of [CNTs–MM–hemin] cathode has rarely been reported in recent literatures related to Li–S batteries (as compared in Table S2 in the Supporting Information). By contrast, the CNTs–MM–hemin and CNT cathodes only provide initial discharge capacities of 899 and 681 mAh g^−1^, dropping rapidly to 350 (capacity decay ratio: 0.068%) and 88 mAh g^−1^ (capacity decay ratio: 0.098%) after 900 cycles, respectively (Figure 3c; Figure S16, Supporting Information). It is further confirmed that [CNTs–MM–hemin] with a periodic covalent network structure plays an important role in accelerating surface reaction kinetics. When increasing the sulfur mass loading to ≈4.1 mg cm^−2^, the [CNTs–MM–hemin] cathode exhibits a high capacity retention of 87% and an areal capacity of 2.14 mAh cm^−2^ over 200 cycles (Figure 3d). Even at the ultrahigh sulfur mass loading of 7.5 mg cm^−2^, the [CNTs–MM–hemin] cathode still reveals the high capacity retention of 83% after 110 cycles (Figure S17, Supporting Information), further demonstrating the long cycle lifespan of [CNTs–MM–hemin] cathode and its potentials in practical application of Li–S batteries.

Cycle‐dependent UV–vis investigations of different composites in electrolyte solution were performed, aiming to find out the underlying reasons for the excellent cycling performance of the [CNTs–MM–hemin] cathode, as shown in Figure S18 in the Supporting Information. It shows that the [CNTs–MM–hemin] sample exhibits the lowest hemin concentration in solution during cycling, clearly suggesting the [CNTs–MM–hemin] possesses the most robust immobilization capability to hemin through the multiple (covalent, coordinated, physical) connections. On the contrary, the significant increases in the hemin concentration in solution were observed in CNTs–MM–hemin and CNTs–hemin during cycling, implying a weak immobilization ability of CNTs–MM–hemin and CNTs–hemin to hemin only through the physical connection (*π*–*π* interaction). The results were further confirmed by a high Fe retention rate (≈92%) in [CNTs–MM–hemin] cathode and a low Fe retention rate (≈53.0% and 29.5%, respectively) in CNTs–MM–hemin and CNTs–hemin cathodes after 500 cycles in inductively coupled plasma‐mass spectrometry (ICP‐MS) analysis (Figure S19, Supporting Information). The positive role of the [CNTs–MM–hemin] in high sulfur utilization and enhanced reaction kinetics was further verified in the electrochemical impedance spectra (EIS) measurement. These spectra in Figure S20a–e in the Supporting Information were analyzed using the equivalent circuit model in Figure S20f in the Supporting Information, where *R*_e_ is electrolyte resistance, *R*_ct_ is interfacial charge transfer resistance, and *R*_mt_ is the mass transportation resistance of the solid‐state layer of the accumulated insoluble Li_2_S_2_/Li_2_S, respectively.^[^ ^32^, ^33^ ^]^ In Figure S21a in the Supporting Information, the [CNTs–MM–hemin] cathode shows the lowest *R*_e_ among all the cathodes over cycling, indicating the smallest dissolution of hemin and LiPS into bulk electrolyte owing to the good immobilization effect of [CNTs–MM–hemin] composite to hemin. Meanwhile, the most stable and lowest *R*_ct_ (Figure S21b, Supporting Information) and *R*_mt_ (Figure S21c, Supporting Information) are also observed in [CNTs–MM–hemin] cathode, demonstrating sustainable and efficient charge/mass transportation for sulfur redox reactions during long‐term cycling.^[^ ^34^ ^]^

### 2.4 Catalytic Mechanisms

**Figure 4:**
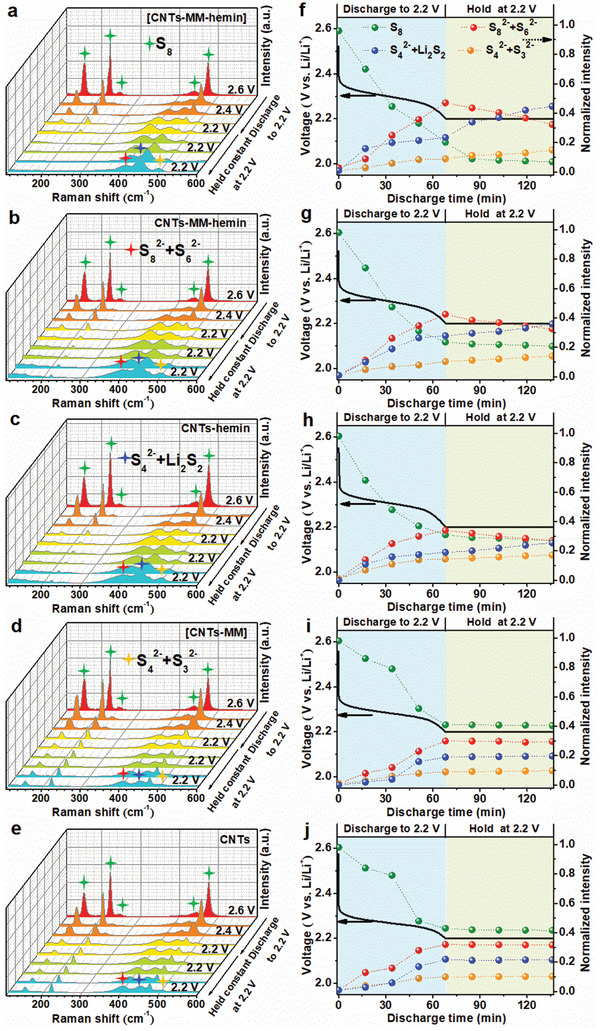
In situ Raman investigation of different cathodes during discharge. In situ Raman spectra of the a) [CNTs–MM–hemin], b) CNTs–MM–hemin, c) CNTs–hemin, d) [CNTs–MM], and e) [CNTs–MM] cathodes during the second discharge, where the cell potentials were scanned from 2.6 to 2.2 V, and kept at 2.2 V for a long time. Time dependence of the normalized peak intensity of different sulfur species at the f) [CNTs–MM–hemin], g) CNTs–MM–hemin, h) CNTs–hemin, i) [CNTs–MM], and j) [CNTs–MM] cathodes.

To gain insight into the catalytic effect of the composite cathode on mitigating LiPS shuttle at the microscale, the Raman spectra were measured in situ during the second discharge of the five cathodes, as shown in **Figure** **4**a–e. The potential dependences of the peak intensities are provided in Figure 4f–j. As the cathodes are discharged from 2.6 to 2.2 V, the intensity depletion of the strong S_8_ peaks (pointed by green stars)^[^ ^35^ ^]^ and the intensity enhancement of long‐chain LiPS (Li_2_S_8_/Li_2_S_6_) peaks (pointed by red stars)^[^ ^36^ ^]^ are observed on the five cathodes, signifying the S_8_ rings are opened to form a large quantity of long‐chain LiPSs. Subsequently, the cell potential was held at 2.2 V, where long‐chain LiPSs are further reduced to short‐chain LiPSs (Li_2_S_4_/Li_2_S_3_/Li_2_S_2_), and Raman spectra from the cathodes were acquired for each 15 min in order to reveal the role of catalysts in suppressing the LiPS migration. There is no much intensity change of both long‐chain and short‐chain LiPS peaks observed with increasing time for [CNTs–MM] and CNT cathodes, but the cathodes with hemin (CNTs–MM–hemin, CNTs–hemin, and [CNTs–MM–hemin] cathode) exhibit a progressive intensity increase of short‐chain LiPS peaks and a considerable intensity loss of long‐chain LiPS peaks during the time, particularly [CNTs–MM–hemin] cathode shows the largest intensity variation. These results clearly evident that hemin can effectively mitigate LiPS shuttle through catalyzing long‐chain LiPSs to short‐chain LiPSs (Li_2_S_8_/Li_2_S_6_ + *n*Li^+^ + *n*e^−^ → Li_2_S_4_/Li_2_S_3_/Li_2_S_2_), especially in the FeN_5_ configuration in the [CNTs–MM–hemin] cathode. The findings correspond well to the symmetric cell (Figure S10, Supporting Information) and in situ UV–vis absorption spectroscopy results (Figure 2f–h; Figures S11–S13, Supporting Information).

Beside the evolution micromechanism of the adsorbed LiPSs, the structural dynamics of the cathode material itself during discharge is also a noteworthy issue. As shown in Figure S22a in the Supporting Information, the Fe 2p_1/2_ and Fe 2p_3/2_ peaks due to hemin in [CNTs–MM–hemin] cathode gradually shift to lower binding energy upon discharge, which suggests that Fe in hemin is gaining electron density from both S atom of LiPSs and N atom of hemin or MM,^[^ ^34^ ^]^ giving rise to stronger Fe—S and Fe—N bonds, but weaker S—S and C—N bond in molecules. As a result, the S—S bond in long‐chain LiPSs easily breaks to form short‐chain LiPSs, and the C–N Raman peak shifts to a lower wavenumber during the sulfur reduction reaction on [CNTs–MM–hemin] cathode (Figure S22b,c, Supporting Information).

The catalytic abilities toward short‐chain LiPS conversion of the aforementioned catalysts were further evaluated by the potentiostatic Li_2_S deposition measurements. As shown in Figure S23 in the Supporting Information, the amounts of the Li_2_S precipitation on the electrodes are in the order of [CNTs–MM–hemin] (261.4 mAh g^−1^) > CNTs–MM–hemin (221.7 mAh g^−1^) > [CNTs–MM] (194.7 mAh g^−1^) > CNTs–hemin (170.5 mAh g^−1^) > CNTs (132.8 mAh g^−1^), whereas their precipitation time is totally in a reverse order ([CNTs–MM–hemin] (6744 s) < CNTs–MM–hemin (7146 s) < CNTs–hemin (8236 s) < [CNTs–MM] (9032 s) < CNTs (10650 s)), revealing the most vigorous Li_2_S nucleation kinetics on [CNTs–MM–hemin] owing to a synergistic catalytic effect of covalent, coordinated, and physical interactions in the system on the conversion of short‐chain LiPSs.^[^ ^37^ ^]^

**Figure 5:**
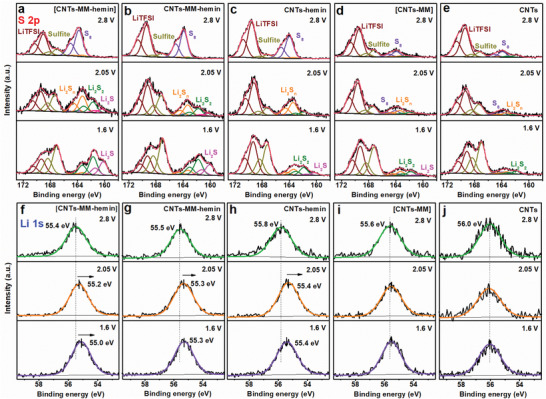
Semi‐in situ S 2p and Li 1s XPS spectra. Semi‐in situ S 2p XPS spectra of a) [CNTs–MM–hemin], b) CNTs–MM–hemin, c) CNTs–hemin, d) [CNTs–MM], and e) CNT cathodes after discharging to specific states. Semi‐in situ Li 1s XPS spectra of f) [CNTs–MM–hemin], g) CNTs–MM–hemin, h) CNTs–hemin, i) [CNTs–MM], and j) CNT cathodes after discharging to specific states.

The reaction mechanisms of [CNTs–MM–hemin] catalysts were also clarified by a semi‐in situ XPS study. The S 2p signals of the Li salts and their decomposition products in different degree are observed at 167.0–170.5 eV^[^ ^38^, ^39^ ^]^ on all the cathodes of discharge states, since the Li salts were not removed from the extracted cathodes surface in order to obtain the full information of the sulfur species (**Figure** **5**a–e). At fully charged state (2.8 V), the S_8_ signals dominate the low binding energies of all the cathodes. After discharging to 2.05 V, the S_8_ signals are severely attenuated and the LiPS (Li_2_S*_n_*, 4 ≤ *n* ≤ 8) signals are enhanced prominently. When the cathodes are fully discharged to 1.6 V, the reduction of Li_2_S*_n_* (4 ≤ *n* ≤ 8) signals and the increase of Li_2_S_2_/Li_2_S signals are observed at the five cathodes. The findings are the typical reduction process of S_8_ to form Li_2_S*_n_* (4 ≤ *n* ≤ 8) then to Li_2_S_2_/Li_2_S on the cathode. On careful analysis, the cathodes containing hemin ([CNTs–MM–hemin], CNTs–MM–hemin, and CNTs–hemin) exhibit the stronger S_8_ and Li_2_S*_n_* (4 ≤ *n* ≤ 8) signals at 2.8 and 2.05 V, respectively, than the hemin‐free cathodes ([CNTs–MM] and CNTs), substantiating the good catalytic effect of hemin on long‐chain LiPS conversion again.^[^ ^40^ ^]^ Moreover, the cathodes with MM ([CNTs–MM] or CNTs–MM–hemin) display larger proportions of Li_2_S_2_/Li_2_S than the MM‐free cathodes (CNTs or CNTs–hemin) at 1.6 V, suggesting MM can catalyze the conversion from Li_2_S*_n_* (4 ≤ *n* ≤ 8) to insoluble Li_2_S_2_/Li_2_S. Upon the catalytic effect of both hemin and MM along with a special FeN_5_ configuration, the [CNTs–MM–hemin] cathode exhibits the strongest Li_2_S_2_/Li_2_S signals and the smallest proportions of Li_2_S*_n_* (4 ≤ *n* ≤ 8) at 1.6 V, manifesting its extraordinary ability to catalyze the LiPS conversion, in good accordance with the foregoing in situ Raman (Figure S22, Supporting Information) and Li_2_S nucleation results (Figure S23, Supporting Information). 

**Figure 6:**
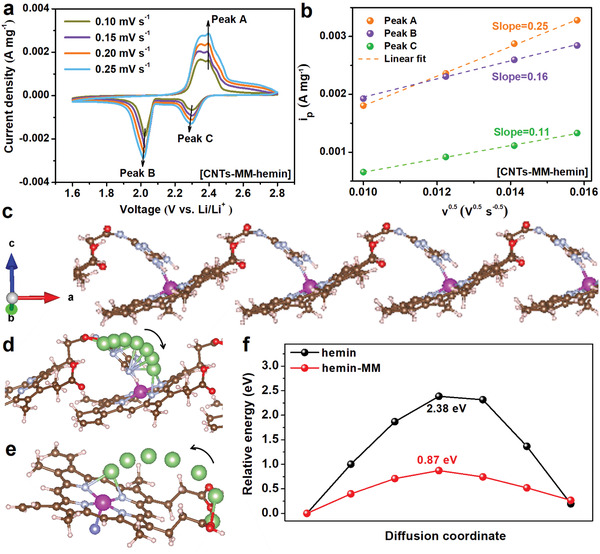
Li^+^ diffusion study. a) CV curves of [CNTs–MM–hemin] cathode at different scan rates. b) Corresponding linear fits of the peak current in panel (a) based on Equation (1). c) Side view of periodic hemin–MM configuration. Side views of Li^+^ migration routes on d) periodic hemin–MM and e) hemin surfaces. f) The relevant energy barrier of Li^+^ diffusion on periodic hemin–MM and hemin surfaces. The light blue, brown, pink, rose pink, red, blue, and green balls are N, C, H, Fe, O, Cl, and Li atoms, respectively.

The Li element is further visualized by the signals at binding energy of ≈55.5 eV in the XPS spectra (Figure 5f–j). At fully charged state (2.8 V), the Li 1s signal due to Li—S bond of LiPSs from pristine CNTs is at slightly higher binding energy than other four cases, indicating less electron loss in other cases, which in turn suggests the electron from N of hemin or MM might transfer to Li^+^, forming a “Li‐bond‐like” S—Li···N bridging configuration. One thing to be noted is that the other four Li 1s peaks are located at different binding energies, which is probably attributed to the discrepancy in forming Li···N bond or S—Li···N bridging configuration between Li^+^ and different N species in various catalytic molecules, in good agreement with the ^7^Li NMR results (Figure 2e). As the cathode is discharged to 2.05 V, the significant shifting toward lower binding energy in Li 1s signal is witnessed at CNTs–hemin cathode, but the shifting stops when the cathode is further discharged to 1.6 V. This directly proves that hemin artificial enzyme as a catalyst does have the strong catalytic conversion ability to long‐chain LiPSs, but not to short‐chain LiPSs. Almost no Li 1s shifting at [CNTs–MM] surface during discharge is the evidence that MM hardly converts short‐chain LiPSs via Li‐bonds without the aid of hemin for accelerating the conversion of long‐chain LiPSs, although MM is beneficial for the conversion of short‐chain LiPSs (as proved by XPS in Figure 5). Despite both hemin and MM are included in the CNTs–MM–hemin cathode, the extent of shifting at 2.05 V is much more significant than the shifting at 1.6 V, as observed at CNTs–hemin, largely due to the discontinuous and uneven distribution of hemin and MM on the surface of the CNTs, where the long Li^+^ diffusion path from the hemin to the nearby MM surface limits the conversion kinetics of short‐chain LiPSs. In [CNTs–MM–hemin] cathode, MM can not only enhance the catalytic activity of hemin toward long‐chain LiPSs and molecular uniform distribution, but also shorten the electron/Li^+^ migration distance between the two molecules through covalent and coordinated bonds, as a result, a fast and smooth catalytic conversion and nucleation of LiPSs can be realized at the interface. That is why a gradual shifting toward lower binding energy of the Li 1s signal during discharge (Figure 5f) and the highest Li^+^ diffusivity in the redox reaction (**Figure** **6**a,b; Figure S24 and Table S3, Supporting Information) are detected at [CNTs–MM–hemin] surface.

Given rich and diverse Li···N bonds exist in the system and easily form Li‐bond networks, a periodic hemin–MM configuration was established here, as shown in Figure 6c and Figure S25 in the Supporting Information, where one end of MM molecule attaches to the Fe center atom of hemin via Fe–N_1_ coordination and the other end (amino group) of MM binds with adjacent hemin through forming covalent amide bond. Based on this model, the migration paths of Li^+^ on the surfaces of periodic hemin–MM and hemin were calculated by climbing image nudged elastic band (CI‐NEB) method, respectively, and are given in Figure 6d,e. The calculated energy barrier of Li^+^ along the diffusion coordinate on hemin surface is 2.38 eV (Figure 6f). Surprisingly, it decreases to 0.87 eV for hemin–MM (Figure 6f), where the Li atoms are preferentially adsorbed on O atoms of amide group and N atoms of MM and form a large number of Li‐bonds (Li—N and Li—O bonds), even Li‐bond networks. The results demonstrate that the multiple Li‐bonds and Li‐bond networks are favorable for reducing the energy barrier of Li^+^ diffusion and enhancing the nucleation kinetics of LiPSs, which is confirmed by the above Li_2_S deposition, Li 1s XPS and Li^+^ diffusivity experiments.

The above analysis can give an overall picture of what is happening in this system. Hemin artificial enzyme inherits strong adsorption and catalytic activity to long‐chain LiPSs via Li···N bond and Fe—S coordinated bond. In the process of discharge, hemin fights for electrons from long‐chain LiPSs through Fe—S bond, promoting the cleavage of the S—S bond in long‐chain LiPSs and the generation of the short‐chain LiPSs. With the help of physical *π*–*π* interactions between the CNTs and the molecules (hemin and MM), amide bonds between MM and hemin/CNTs, and Fe–N coordination between hemin and MM, MM cofactor not only acts as a stabilizer for easy‐lost hemin, but also has high adsorption ability to Li^+^ for a fast solid Li_2_S nucleation. The foregoing short‐chain LiPSs trapped on hemin surface rapidly diffuse to adjacent MM and form abundant and various Li‐bonds (multiple Li···N bonds), even Li‐bond networks, between them, accelerating electron/Li^+^ transfer in the system and boosting the conversion of short‐chain LiPSs to insoluble Li_2_S_2_/Li_2_S. With the synergistic effect of covalent, coordinated and physical interactions between hemin, MM and CNTs, the [CNTs–MM–hemin] catalyst guarantees the smooth sulfur catalytic conversion to mitigate the shuttle effect of LiPSs effectively and improve the electrochemical performance of Li–S batteries.

## 3 Conclusion

In conclusion, a new cofactor‐assisted artificial enzyme with multiple Li‐bond networks, i.e., [CNTs–hemin–MM], was proposed to accelerate the SRR kinetics and suppress the polysulfide shuttle in Li–S batteries. A large number of well‐dispersed FeN_5_ sites as catalytic active centers of hemin and perfect Li‐bond electronic transmission networks offered by MM cofactors on CNT conductive substrates fully realize the efficient and stable catalytic superiorities to promote both long‐chain and short‐chain LiPS conversions. Comprehensive spectroscopic and electrochemical characterizations combined with theoretical calculations elaborate the catalytic effects and reaction mechanisms on LiPS conversion at [CNTs–hemin–MM] cathodes, particularly the synergistic functions of the covalent amide bonds, coordinated FeN_5_ configurations and physical *π*–*π* conjugations formed between hemin, MM, and CNTs. The high and sustained catalytic effects toward LiPSs decide outstanding electrochemical performances of Li–S batteries, exhibiting impressive stability and high sulfur loading performance. The present work opens up a new and sustainable avenue on the design of cofactor‐assisted artificial enzyme and multiple Li‐bond networks to achieve efficient sulfur conversion for advanced rechargeable lithium batteries. It is believed that the concept of multiple Li‐bond networks may also inspire the understanding of Li^+^ transport properties and growth behavior of Li dendrites and propel the development of high‐performance lithium secondary battery systems.

## 4 Experimental Section

### Material Synthesis

[CNTs–MM–hemin] composite was fabricated using the previously reported method.^[^ ^41^ ^]^ First, 10 mg hemin (>95%, Alfa Aesar) and 10 mg carboxylated multiwalled CNTs (>99.9%, inside diameter: 3–5 nm, Aladdin) were dispersed in anhydrous ethanol (5 mL, >99.5%, Aladdin) under ultrasonication. The 7 mg 1‐ethyl‐3(3‐dimethylaminopropyl) carbodiimide (EDC, >97%, Sigma‐Aldrich) and 7 mg *N*‐hydroxysulfosuccinimide sodium salt (Sulfo‐NHS, >98%, Sigma‐Aldrich) were further injected into the above mixture and stirred for 2 h to activate the carboxylic groups of hemin and CNTs. Subsequently, a MM (>99%, Alfa Aesar) solution (10 mg of MM dissolved in 3 mL of anhydrous ethanol) was introduced into the mixture and stirred overnight for producing amide bonds by a condensation reaction between the carboxylic acid group of hemin/CNTs and the amino groups of MM. The obtained dispersion was then centrifuged at 3000 rpm for 5 min. This procedure was repeated three times. The resulting wet [CNTs–MM–hemin] composite was allowed to evaporate at 55 ℃ in vacuum to dryness. The average Fe content in [CNTs–MM–hemin] composite was measured to be 2.61 wt% by ICP‐MS (iCAP Qc, Thermo Fisher Scientific), indicating that ≈91% of hemin molecules was effectively attached onto the CNTs. For comparison, the composite without hemin (denoted as [CNTs–MM]) was fabricated under the same condition of [CNTs–MM–hemin]. The composites without condensation method (denoted as CNTs–MM–hemin and CNTs–hemin, in which hemin is physically bonded to its host) were fabricated by ultrasonicating CNTs, hemin, and MM in a mass ratio of 1:1:1 or CNTs and hemin in a mass ratio of 1:1 in *N*‐methyl‐2‐pyrrolidinone (NMP, >99.5%, Aladdin) followed by hot drying treatment at 55 °C for 8 h.

### Material Characterization

HAADF‐STEM images and EDX elemental mappings of [CNTs–MM–hemin] were collected at 200 kV using a FEI‐Themis Z instrument equipped with a probe corrector, a high‐angle annular dark‐field detector, and EDX detector. Thermo Fisher Scientific K‐Alpha instrument served as X‐ray photoelectron spectrometer (Excitation source: Al K*α*). FTIR spectra were recorded using a Fourier transform infrared spectrometer (iS50, Thermo Fisher Scientific). The concentration of hemin dissolved in electrolyte was investigated by a UV–vis spectrophotometer (UV‐1800, Shimadzu). ^7^Li high‐resolution solid‐state NMR spectra were recorded on an Agilent 600 M spectrometer with a standard Agilent 4 mm magic angle spinning (MAS) probe. The Fe content in [CNTs–MM–hemin] composite was determined by the ICP‐MS (iCAP Qc, Thermo Fisher Scientific).

### Visualized Adsorption of Li_2_S_6_

Li_2_S_6_ solution (0.015 m) was obtained by dissolving Li_2_S (>99.98%, Aladdin) and S (>99.99%, Aladdin) with a molar ratio of 1:5 in a mixed solvent of DME (>99.95%, Suzhou Dodo Chem. Ltd.)/DOL (>99.95%, Suzhou Dodo Chem. Ltd.) (v/v = 1:1) under stirring at 50 °C for 24 h in Ar atmosphere. To evaluate the sulfur adsorption ability of the composites, 20 mg of [CNTs–MM–hemin], [CNTs–MM], and CNTs were immersed into the 2 mL of Li_2_S_6_ solutions, respectively. The digital photographs were collected after 24 h.

### In Situ UV–Vis Spectroscopic Measurements

The configuration of the spectroelectrochemical cell used for in situ UV–vis analysis was reported in the previous work,^[^ ^42^ ^]^ where the [CNTs–MM–hemin]‐coated glassy carbon (GC), platinum wire, and sealed Ag/AgCl were used as working electrode, counter electrode, and reference electrode, respectively. The 0.5 × 10^−3^ m Li_2_S*_n_* (*n* = 8, 6, 4) solutions were used as electrolytes, which were prepared by mixing Li_2_S and S powder with the stoichiometric molar ratio of 1:*x* (*x* = 7, 5, 3) into dimethyl sulfoxide (DMSO, >99.9%, Aladdin) and stirring for 24 h in Ar atmosphere. For in situ UV–vis measurements, the cell was attached to a UV–vis spectrometer (UV‐1800, Shimadzu). In this way, the UV beam could directly focus on the glass of the cell. During the CVs of the cell were testing between 1.6 and 2.8 V with a scanning rate of 0.4 mV s^−1^, the UV–vis spectra were recorded with a potential interval of 0.1 V. To clearly demonstrate the changes in the amount of soluble LiPSs during discharge, the UV–vis absorbance was normalized by making the absorbance at 2.8 V as 1 and the normalized intensities of the absorbance peaks for S_8_^2−^ (≈492 nm), S_6_^2−^ (≈475 nm), S_4_^2−^ (≈420 nm), and S_3_^*−^ (≈617 nm) were plotted as a function of potential. For comparison, the working electrode coated with CNTs–hemin, [CNTs–MM], and CNTs were also measured in the same way.

### Fabrication of [CNTs–MM–Hemin], CNTs–MM–Hemin, CNTs–Hemin, [CNTs–MM], and CNT Cathodes

The multiwalled carbon nanotubes–sulfur (CNTs–S) composites were fabricated by a melt‐diffusion strategy. The CNTs were mixed with S powders at a mass ratio of 1:4 by sufficient grinding. The mixture was dispersed in carbon disulfide (CS_2_, >99.9%, Aladdin) and thoroughly stirred, and then annealed at 155 °C for 12 h under Ar atmosphere. After cooling, the CNTs–S composites were obtained. The sulfur contents of CNT–S composites in this work were usually controlled between 75 and 80 wt%. The [CNTs–MM–hemin] cathode was prepared by coating the mixed slurry of 80 wt% CNTs–S composites, 5 wt% polyvinylidene fluoride (PVDF, Sigma‐Aldrich), 13 wt% conductive agent, and 2 wt% [CNTs–MM–hemin] composites in NMP on the Al foil. The cathode was then dried at 55 °C overnight in a vacuum oven and cut into a disk with a diameter of 14 mm. The low areal mass loading of sulfur in this study was around 1.4 mg cm^−2^, while the high sulfur loading was 4.1 and 7.5 mg cm^−2^. The preparation of [CNTs–MM], CNTs–MM–hemin, CNTs–hemin, and CNT cathodes was under the same condition, except for replacing the [CNTs–MM–hemin] composites by [CNTs–MM], CNTs–MM–hemin, CNTs–hemin, and CNTs, respectively.

### Electrochemical Measurements

As‐prepared cathode, Celgard 2400 polypropylene (PP) separator, and lithium metal anode were assembled into coin cells (CR2025) in an Ar‐filled glovebox, where the oxygen and moisture contents were kept below 0.1 ppm. The electrolyte was 1.0 m lithium bis (trifluoromethane) sulfonimide (LiTFSI, >99.95%, Sigma‐Aldrich) in a mixed solvent of DME/DOL (v/v = 1:1) with 1 wt% anhydrous LiNO_3_ additive. The electrolyte/sulfur ratio for the coin cell was fixed at 15:1 *µ*L mg^−1^. The electrode area was ≈1.53 cm^−2^. Considering the experimental reproducibility, 10 cells were prepared and tested under the same operating conditions. Cyclic voltammetry was recorded at a scan rate of 0.05–0.25 mV s^−1^ in the potential range of 1.6–2.8 V (vs Li/Li^+^) on an electrochemical workstation (CHI 760E, Chenhua Instruments). EIS measurements were carried out over a frequency range of 100 kHz to 0.01 Hz with an amplitude of 5 mV on the CHI 760E electrochemical workstation. Galvanostatic charge–discharge, rate performance, and cycle life measurements were characterized by a Neware battery testing system (CT‐4008T‐5V20mA‐164, Shen Zhen Netware Technology) between 1.6 and 2.8 V (vs Li/Li^+^) at room temperature. All the electrochemical measurements mentioned previously were conducted at ambient temperature (30 °C). All current and capacity were calculated based on the sulfur mass of the cathode.

### Li_2_S_6_ Symmetrical Cell Assembly and Measurements

The electrodes for symmetrical cells were fabricated in the absence of sulfur according to the previous literature.^[^ ^43^ ^]^ Typically, each material ([CNTs–MM–hemin], CNTs–MM–hemin, CNTs–hemin, [CNTs–MM], or CNTs) was ultrasonically dispersed in NMP and the uniform slurries were formed after vigorously stirring. The slurries were coated on Al foils and dried at 55 °C for 12 h in vacuum. Finally, the electrodes (≈0.4 mg cm^−2^) could be acquired after being punched with a diameter of 14 mm. Two identical electrodes as the working and counter electrodes were assembled into a 2025 coin cell with a Celgard 2400 PP separator and a solution of DME/DOL (v/v = 1:1) containing Li_2_S_6_ (0.25 m) as electrolyte (20 µL). CV curves of Li_2_S_6_ symmetrical cells were carried out in the voltage range from −1.0 to 1.0 V at a scan rate of 50 mV s^−1^ on CHI 760E electrochemical workstation.

### In Situ Raman Spectroscopic Measurements

The cathodes were fabricated by the similar methods as those of the battery performance experiments, except for changing the current collectors as carbon papers (CPs). As shown in Figure S26 in the Supporting Information, the Li–S coin cells with different cathodes, Celgard 2400 PP separators, and Li foil were configured for in situ Raman spectroscopic study. 1.0 m LiTFSI in the mixed solvent of DOL/DME (v/v = 1:1) with 1 wt% LiNO_3_ additive as electrolyte was injected into the cell. A 532 nm laser (laser spot size: ≈1 *µ*m) was passed through the quartz window equipped on the upper case and focused onto the active‐material‐coated cathode side. The Raman spectra were acquired by a Renishaw inVia Raman microscope at different potentials during a negative scanning potential from 2.6 to 2.2 V.

### Nucleation of Li_2_S Study

S and Li_2_S in a molar ratio of 7:1 were stirred in tetraglyme (TEGDME, >98%, Suzhou Dodo Chem. Ltd.) solvent at 50 °C for 24 h to acquire the Li_2_S_8_ solution ([S] = 4 m). The electrode fabrication methods for Li_2_S nucleation study were the same as those of the symmetric cell experiments. 20 µL of Li_2_S_8_ solution was deposited onto the [CNTs–MM–hemin], CNTs–MM–hemin, CNTs–hemin, [CNTs–MM], and CNT electrodes, respectively, while 20 µL of TEGDME without Li_2_S_8_ was dropped onto Li anode. The 2025 coin cells with Celgard 2400 PP separators were first galvanostatically discharged at 112 µA to 2.06 V (vs Li/Li^+^) to consume most long‐chain LiPSs, and then kept potentiostatically at 2.02 V (vs Li/Li^+^) to drive the nucleation and growth of Li_2_S until the current was below 10^−5^ A. The current was recorded during the potentiostatic process. The whole charge was mathematically divided into three parts, including the triangular, rectangular, and peak areas, corresponding to the reduction of Li_2_S_8_ and Li_2_S_6_ and the precipitation of Li_2_S, respectively.

### Semi‐In Situ XPS Measurements

The chemical composition of the cathode surfaces at different discharge potentials was determined by XPS (K‐Alpha, Thermo Fisher Scientific) with a monochromatic Al‐K*α* radiation (10 mA, 15 kV). After ten cycles, the coin cells were discharged to a certain state and disassembled in the Ar‐filled glovebox. The cathodes were extracted from the cells and dried at room temperature overnight in the glovebox, and then quickly transferred in inert gas atmosphere to the XPS vacuum chamber. The spectra were calibrated to the C 1s signal at 284.8 eV and processed with CasaXPS software. For the sake of preserving the holonomic information of active sulfur species, no solvent was employed to clean the cathode surface to remove residual products from the decomposition of the electrolyte. Consequently, the strong S 2p peaks of LiTFSI (169.7 eV/170.8 eV) and sulfites (167.1 eV/168.2 eV) originated from the decomposition of electrolyte were detected in all samples.

### Li^+^ Diffusion Study

The CV tests were also performed at different scan rates to characterize the diffusion coefficient of Li^+^ (*D*_Li_) through Equation (1)

(1) $$i_{p}=\left(2.65\times 10^{5}\right)n^{1.5}S{D_{\mathrm{Li}}}^{0.5}C_{\mathrm{Li}}\nu^{0.5}$$

In this equation, *i*_p_ is the peak current, *n* is the charge transfer number, *S* is the electrode area, *D*_Li_ is the diffusion coefficient of Li^+^, *C*_Li_ is the Li^+^ concentration in the electrolyte, and *ν* is the scan rate. Since *n*, *S*, and *C*_Li_ are constant, *i*_p_ has a linear relation with *ν* ^0.5^ and *D*_Li_ can be obtained from the slopes of curves.

### Computational Methods

DFT calculations were conducted using the Vienna Ab initio Simulation Package (VASP 5.3.5) with the projector augmented wave (PAW) and Perdew–Burke–Ernzerhof (PBE) exchange–correlation functional.^[^ ^44^, ^45^, ^46^, ^47^ ^]^ The plane‐wave cutoff energy was set as 450 eV. For hemin‐based structure optimizations, a 1 × 1 × 1 Monkhorst–Pack (MP) *k*‐point grid was used to sample the Brillouin zone.^[^ ^48^ ^]^ The convergence criterion for the electronic self‐consistent iteration and force was set to 10^−5^ eV and 0.01 eV Å^−1^, respectively. The diffusion barriers for the Li atom over hemin‐based structures were calculated based on the CI‐NEB method.^[^ ^49^, ^50^, ^51^ ^]^ The adsorption energies (*E*_ads_) for LiPSs on surfaces were determined by Equation (2)

(2) $$E_{\mathrm{ads}}=E_{\mathrm{total}}-E_{\mathrm{surf}}-E_{\mathrm{LiPS}}$$

where *E*_total_, *E*_surf_, and *E*_LiPS_ represent the total energies of the adsorbed systems, pristine substrates, and the isolated LiPS clusters, respectively.

## Conflict of Interest

The authors declare no conflict of interest.

## Supporting information

Supporting Information

## Data Availability

Research data are not shared.
