# Supplementary material for: Cofactor‐Assisted Artificial Enzyme with Multiple Li‐Bond Networks for Sustainable Polysulfide Conversion in Lithium–Sulfur Batteries
Source: Adv Sci (Weinh). 2021 Nov 7;9(3):2104205. doi: 10.1002/advs.202104205 (PMC8787425; doi:10.1002/advs.202104205)
Supplement: Supplementary file 1 — Supporting Information [file ADVS-9-2104205-s001.pdf]

ADVANCED SCIENCE
Open Access

Supporting Information

for *Adv. Sci.*, DOI: 10.1002/advs.202104205

Cofactor-Assisted Artificial Enzyme with Multiple Li-Bond Networks for Sustainable Polysulfide Conversion in Lithium–Sulfur Batteries

*Suya Zhou, Shuo Yang, Dong Cai, Ce Liang, Shuang Yu, Yue Hu, Huagui Nie, and Zhi Yang**

Supporting Information

Cofactor-Assisted Artificial Enzyme with Multiple Li-Bond Networks for Sustainable Polysulfide Conversion in Lithium–Sulfur Batteries

*Suya Zhou[#], Shuo Yang[#], Dong Cai, Ce Liang, Shuang Yu, Yue Hu, Huagui Nie, and Zhi Yang**

S. Zhou, Dr. S. Yang, Dr. D. Cai, C. Liang, S. Yu, Dr. Y. Hu, Prof. H. Nie, Prof. Z. Yang

Key Laboratory of Carbon Materials of Zhejiang Province, Wenzhou University, Wenzhou, 325035, China

E-mail: yangzhi@wzu.edu.cn.

Dr. S. Yang

College of Electrical and Electronic Engineering, Wenzhou University, Wenzhou, 325035, China

[#] S.Z. and S.Y. contributed equally to this work

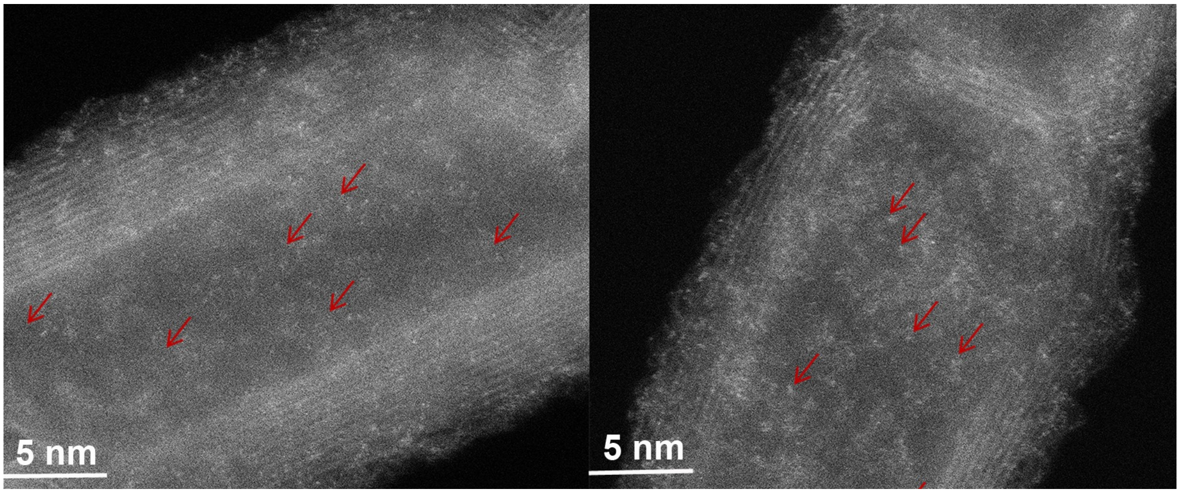

Figure S1. HAADF-STEM images of [CNTs-MM-hemin] composites, in which the atomically-dispersed Fe atoms on the CNTs are pointed by the red arrows.

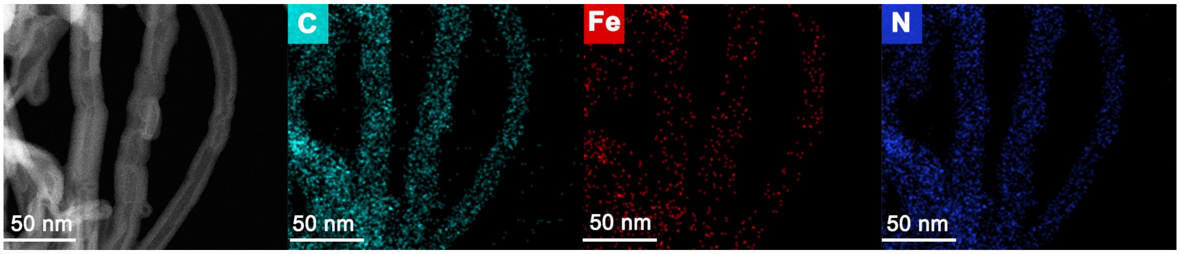

Figure S2. HAADF-STEM image and the corresponding EDX elemental mapping images of C, Fe, N for [CNTs-MM-hemin] composites.

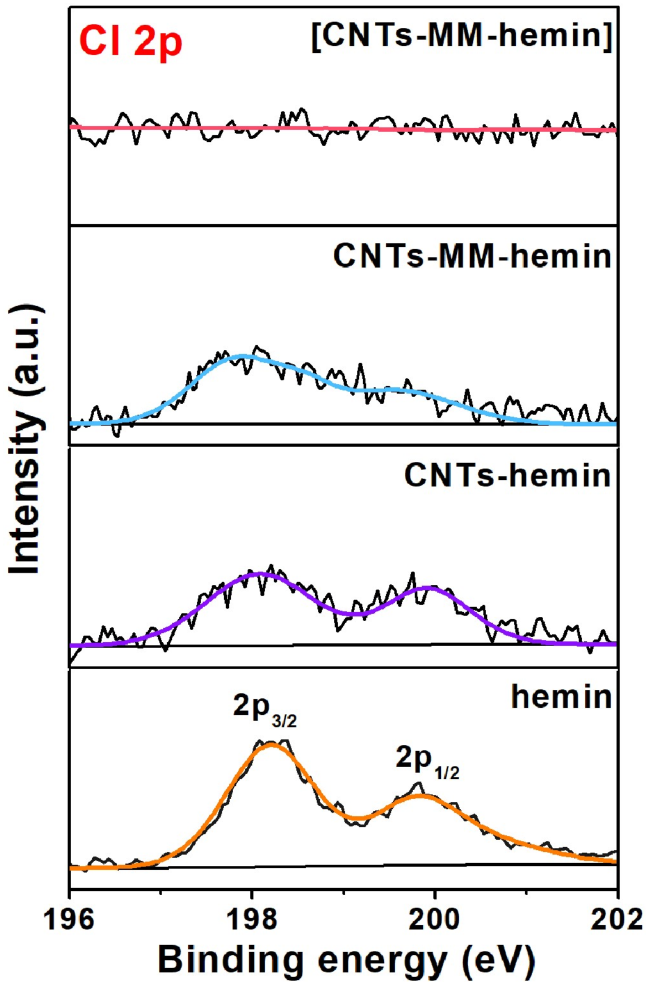

Figure S3. Cl 2p XPS spectra of [CNTs-MM-hemin], CNTs-MM-hemin, CNTs-hemin and hemin.

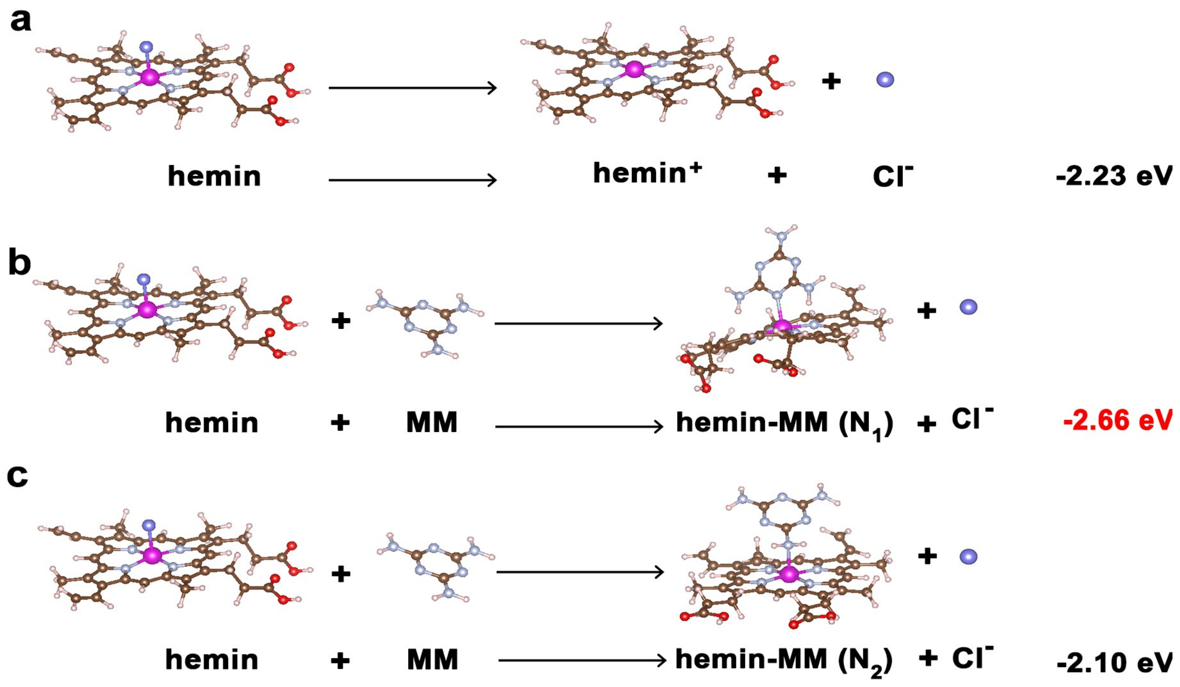

Figure S4. Theoretical calculations of dechlorination reaction energy. a–c) Density functional theory (DFT) calculations of the dechlorination reaction energy in hemin (a), hemin-MM (N_1) (b) and hemin-MM (N_2) (c) systems.

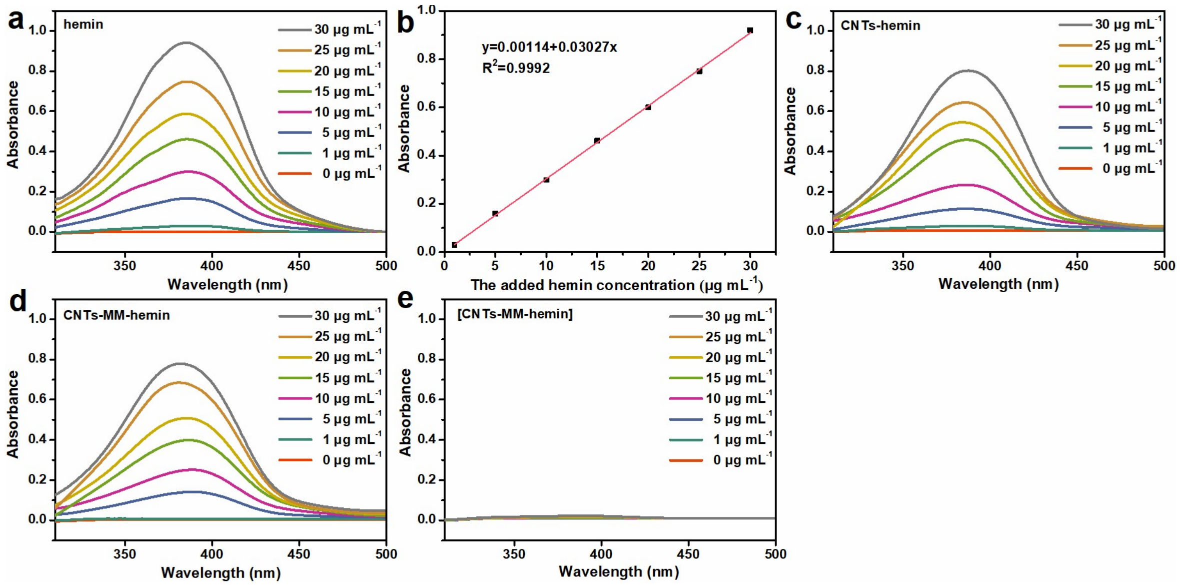

Figure S5. Stability characterization of different composites by UV–vis spectroscopy. a) UV–vis spectra of different concentrations of hemin between 310 nm and 500 nm. b) Calibration curve obtained from data reported in Figure S5a (Supporting Information). c–e) UV–vis spectra of the dissolved hemin in solution at CNTs-hemin (c), CNTs-MM-hemin (d) and [CNTs-MM-hemin] (e) composites with different concentrations of hemin between 310 nm and 500 nm.

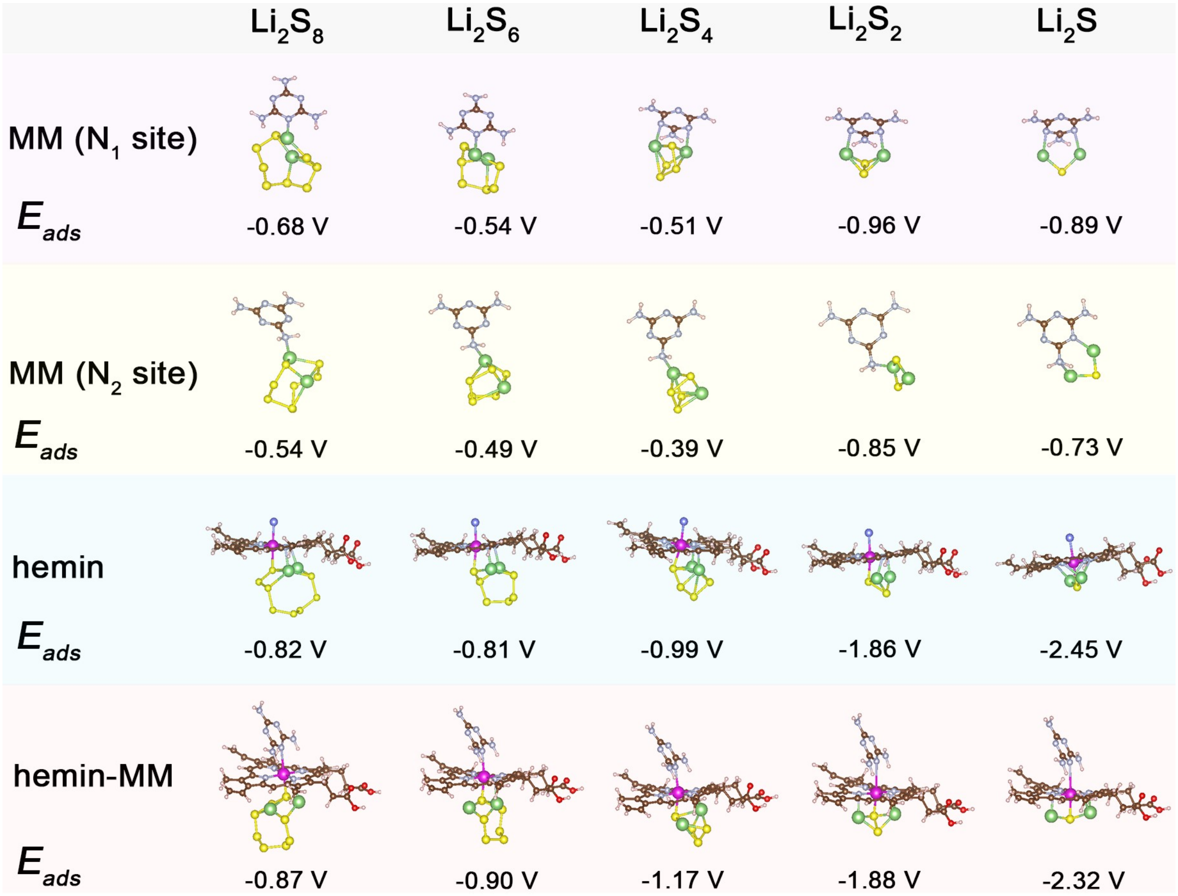

Figure S6. Theoretical calculations of the adsorption energies for LiPSs on different surfaces. The optimized local atomic structure and adsorption energies between Li_2S_n (n = 8, 6, 4, 2, 1) species and hemin-MM, hemin, and MM with N_1 and N_2 site surfaces predicted via DFT calculations. The light blue, brown, pink, rose pink, red, blue, yellow and green balls are N, C, H, Fe, O, Cl, S and Li atoms, respectively.

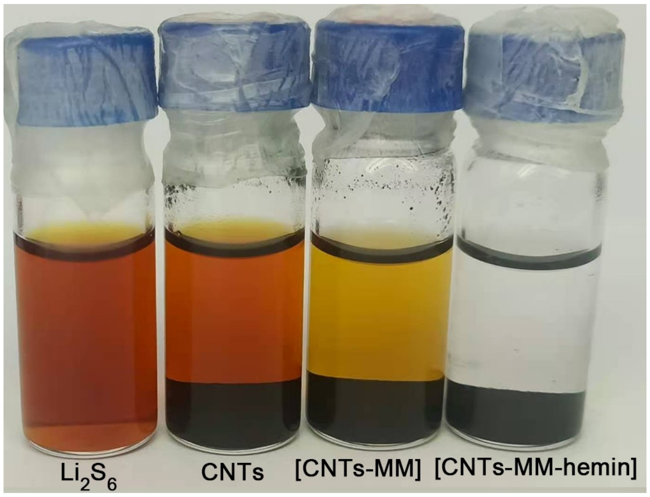

Figure S7. Visualized adsorption tests of Li_2S_6. Polysulfide adsorption tests of CNTs, [CNTs-MM] and [CNTs-MM-hemin] composites.

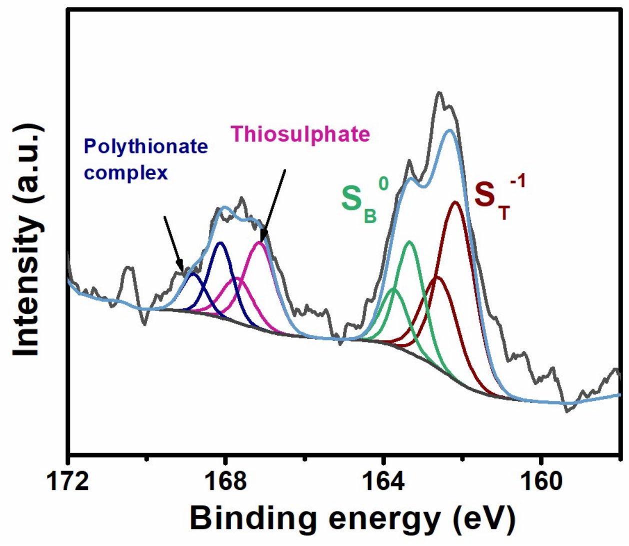

Figure S8. S 2p XPS spectrum of Li_2S_6-treated [CNTs-MM-hemin].

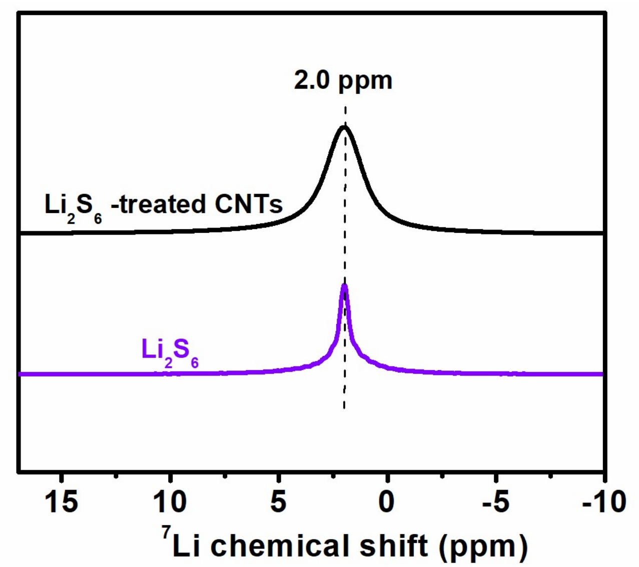

Figure S9. ^{7}Li NMR spectra of Li_2S_6 and Li_2S_6-treated CNTs.

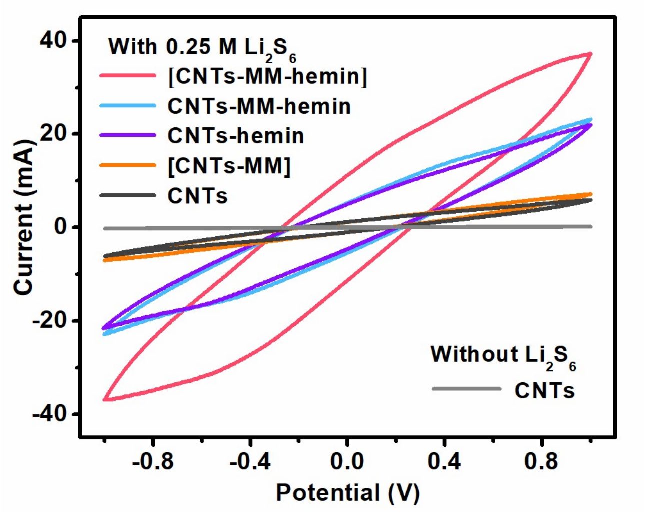

Figure S10. The CV curves of the Li_2S_6 and Li_2S_6-free symmetric cells based on [CNTs-MM-hemin], CNTs-MM-hemin, CNTs-hemin, [CNTs-MM] and CNT electrodes at 50 mV s^{-1}.

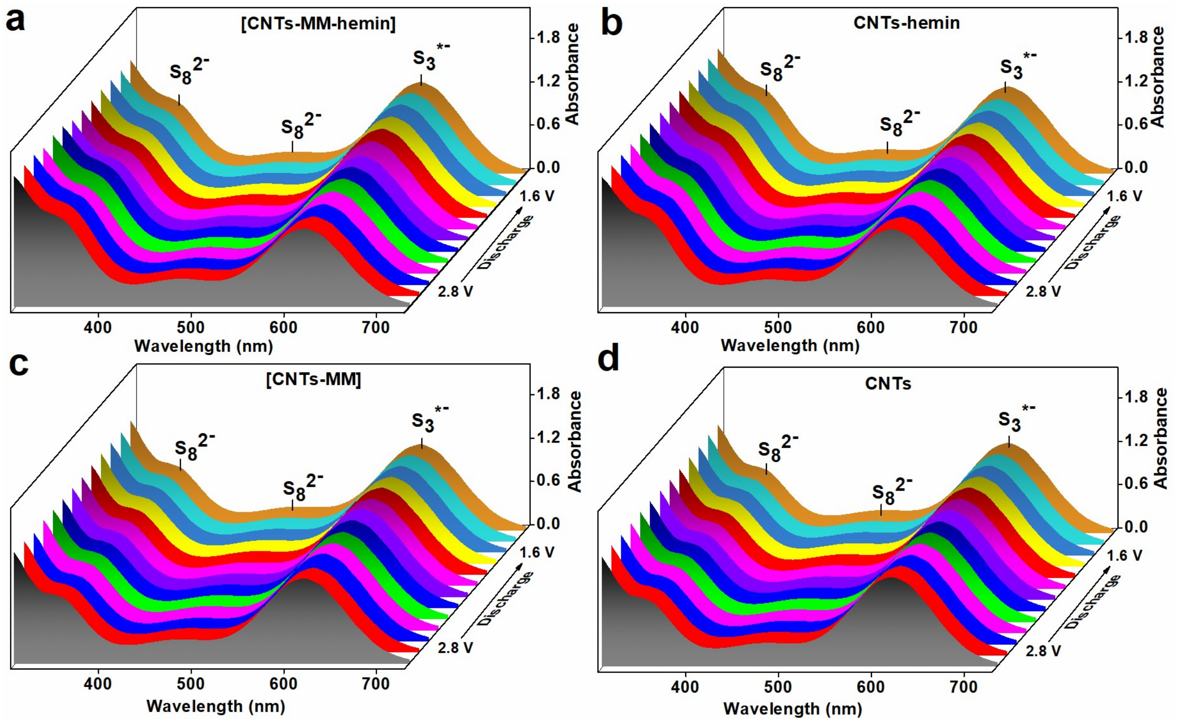

Figure S11. In situ UV–vis absorption spectra of different cathodes in Li_2S_8 solution. a–d) In situ UV–vis absorption spectra of [CNTs-MM-hemin] (a), CNTs-hemin (b), [CNTs-MM] (c) and CNT (d) electrodes in Li_2S_8 solution during discharge process.

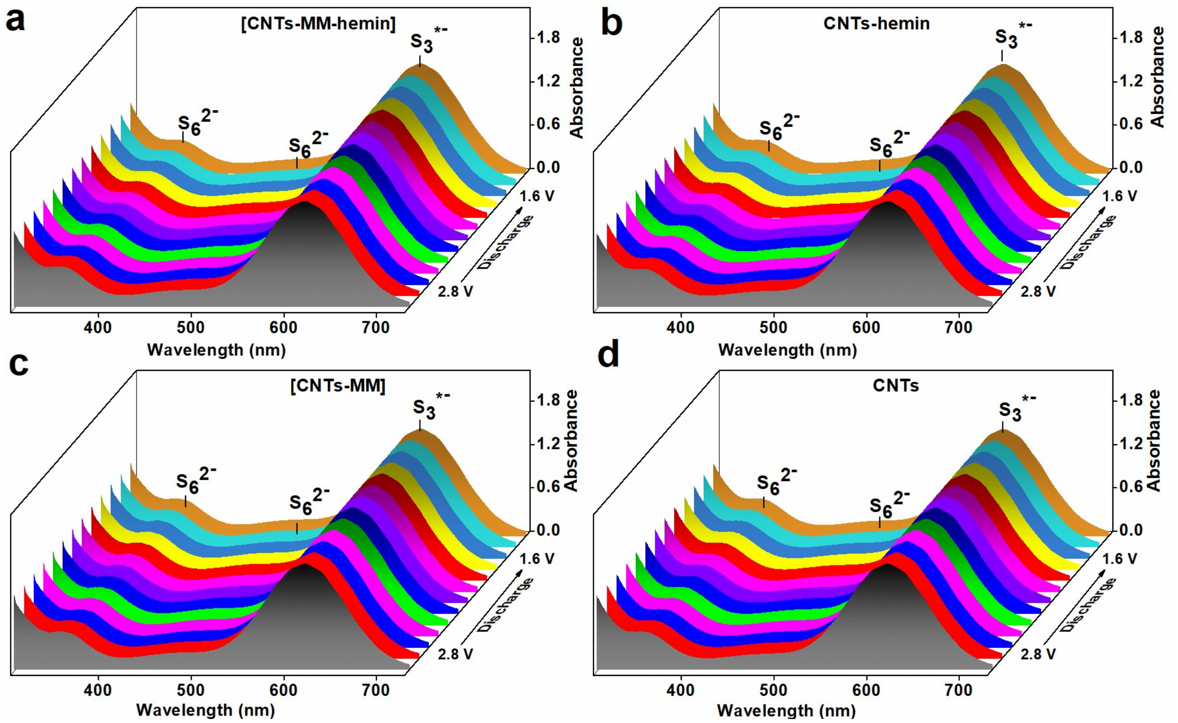

Figure S12. In situ UV–vis absorption spectra of different cathodes in Li_2S_6 solution. a–d) In situ UV–vis absorption spectra of [CNTs-MM-hemin] (a), CNTs-hemin (b), [CNTs-MM] (c) and CNT (d) electrodes in Li_2S_6 solution during discharge process.

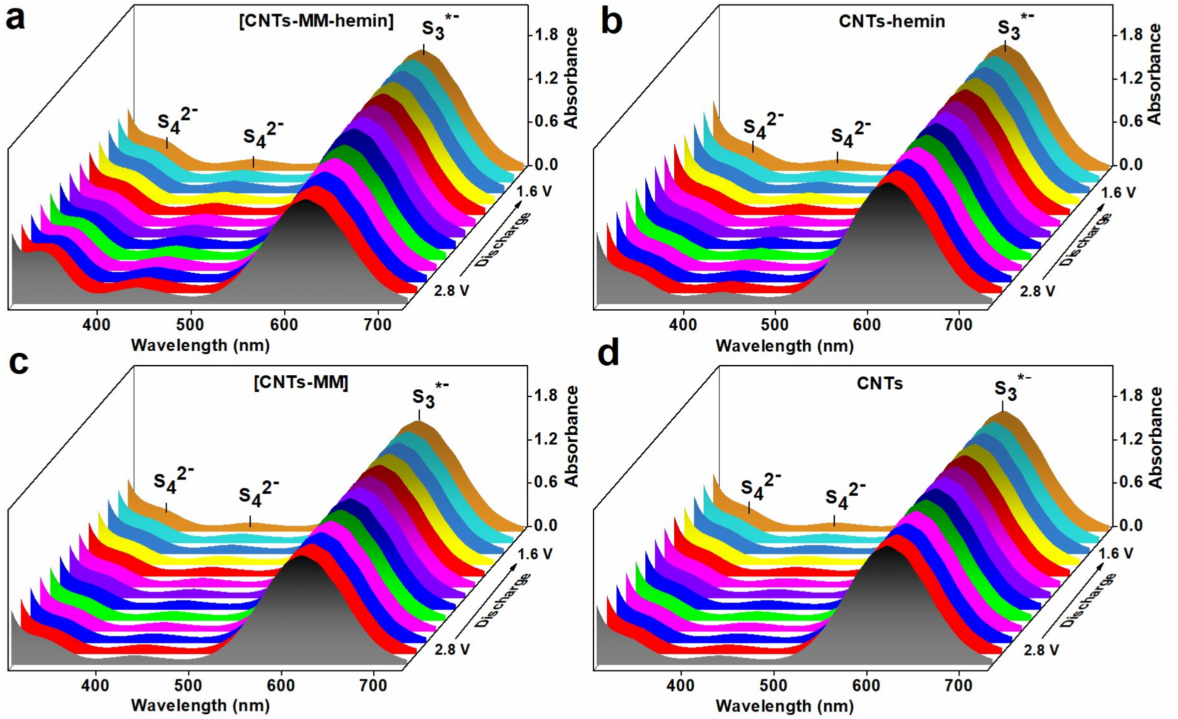

Figure S13. In situ UV–vis absorption spectra of different cathodes in Li_2S_4 solution. a–d) In situ UV–vis absorption spectra of [CNTs-MM-hemin] (a), CNTs-hemin (b), [CNTs-MM] (c) and CNT (d) electrodes in Li_2S_4 solution during discharge process.

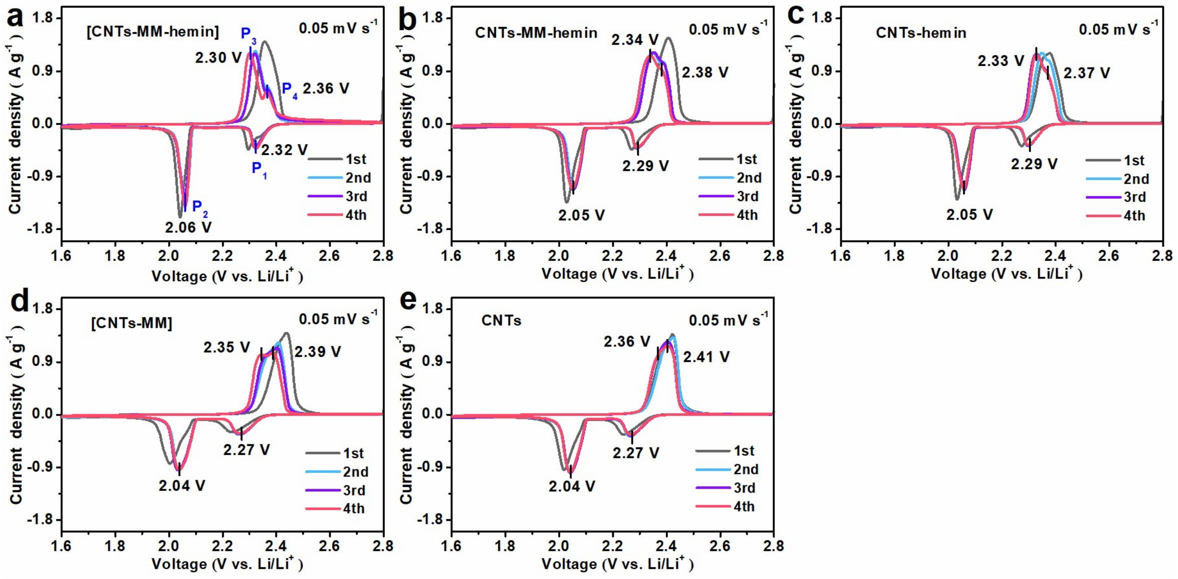

Figure S14. CVs of different cathodes. a–e) CVs of the [CNTs-MM-hemin] (a), CNTs-MM-hemin (b), CNTs-hemin (c), [CNTs-MM] (d) and CNT (e) cathodes between 1.6 and 2.8 V for the first four cycles, respectively, recorded at a scan rate of 0.05 mV s^{-1}.

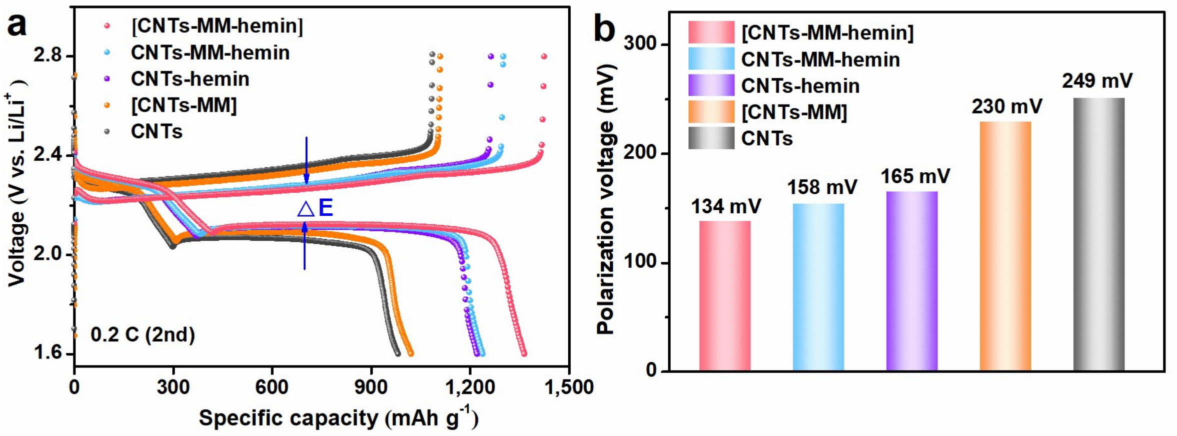

Figure S15. Charge/discharge curves and polarization voltage information. a) Galvanostatic charge/discharge profiles of the [CNTs-MM-hemin], CNTs-MM-hemin, CNTs-hemin, [CNTs-MM] and CNT cathodes at 0.2 C in the second cycle. b) Comparison of polarization voltage (ΔE, the voltage gap between the charge and discharge plateaus) of the five cathodes obtained from the galvanostatic charge/discharge profiles in Figure S14a (Supporting Information).

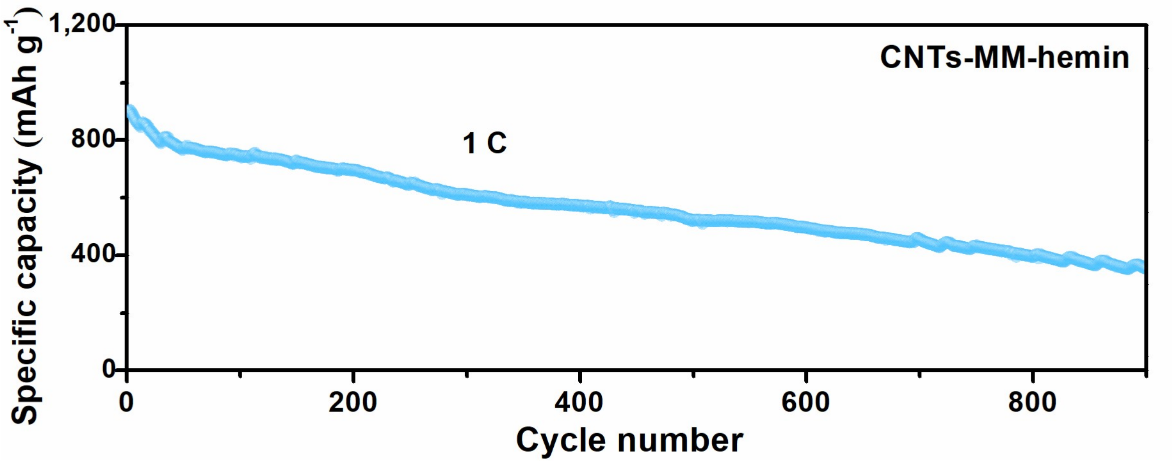

Figure S16. Cycling performance of the CNTs-MM-hemin cathode over 900 cycles at 1 C.

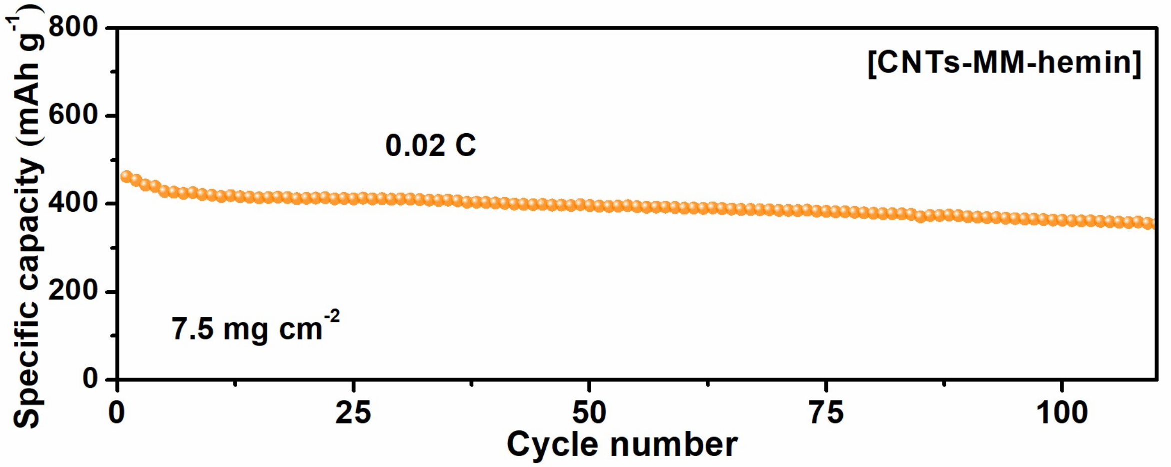

Figure S17. Cycling stability of the [CNTs-MM-hemin] cathode with the sulfur mass loading of 7.5 mg cm^{-2} at 0.02 C for 110 cycles.

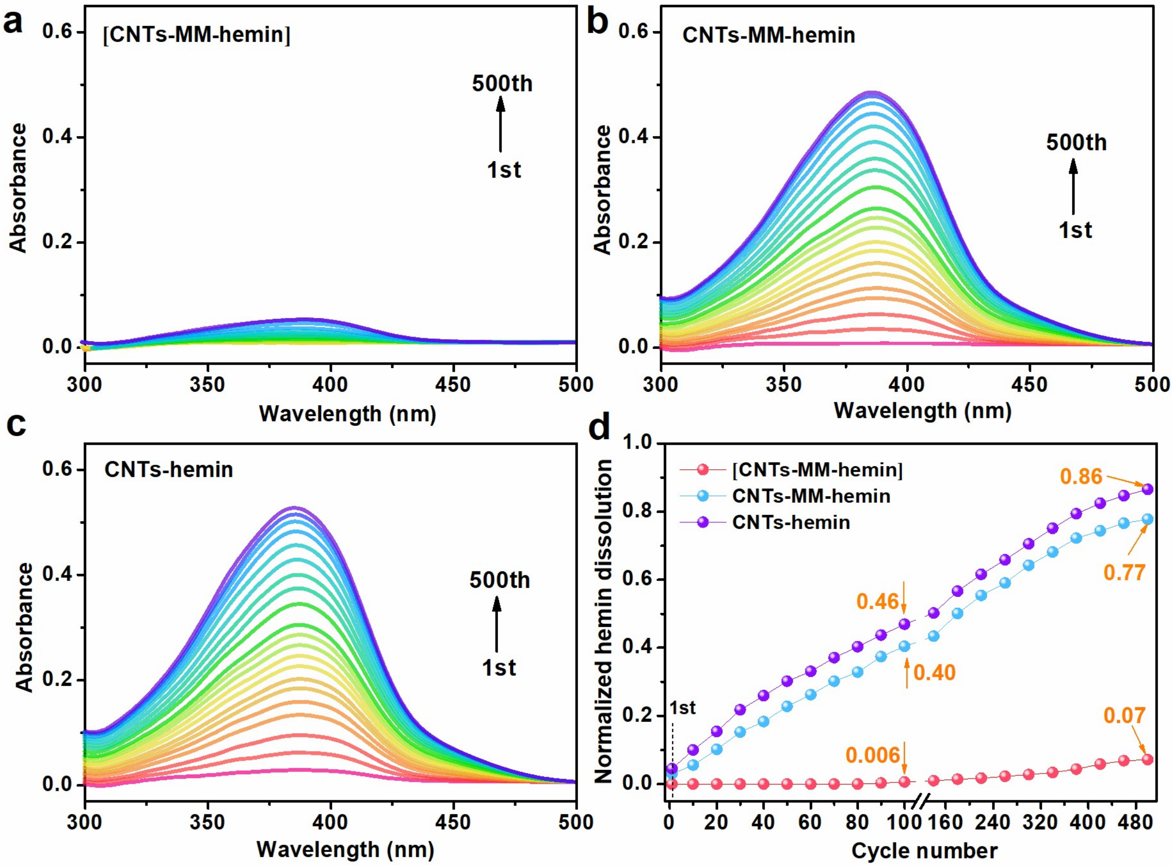

Figure S18. Immobilization capability investigations of different catalysts to hemin by cycle-dependent UV–vis spectroscopy. a–c) UV–vis spectra of hemin in [CNTs-MM-hemin] (a), CNTs-MM-hemin (b) and CNTs-hemin (c) composites after different cycles. d) Solubility curves of hemin in [CNTs-MM-hemin], CNTs-MM-hemin and CNTs-hemin composites during cycling.

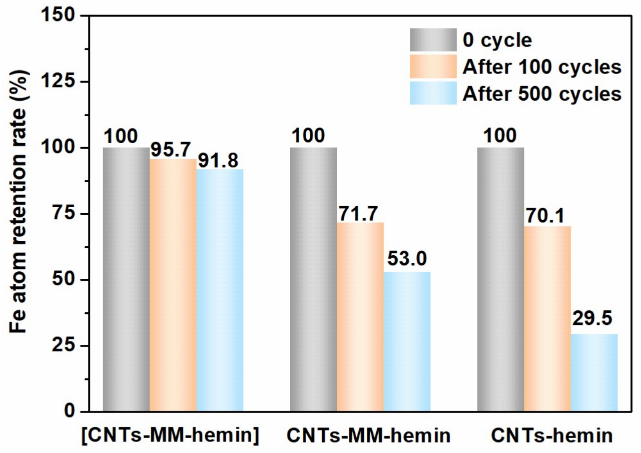

Figure S19. ICP-MS analysis of the Fe content in the cathodes containing [CNTs-MM-hemin], CNTs-MM-hemin and CNTs-hemin composites after 0, 100, 500 cycles.

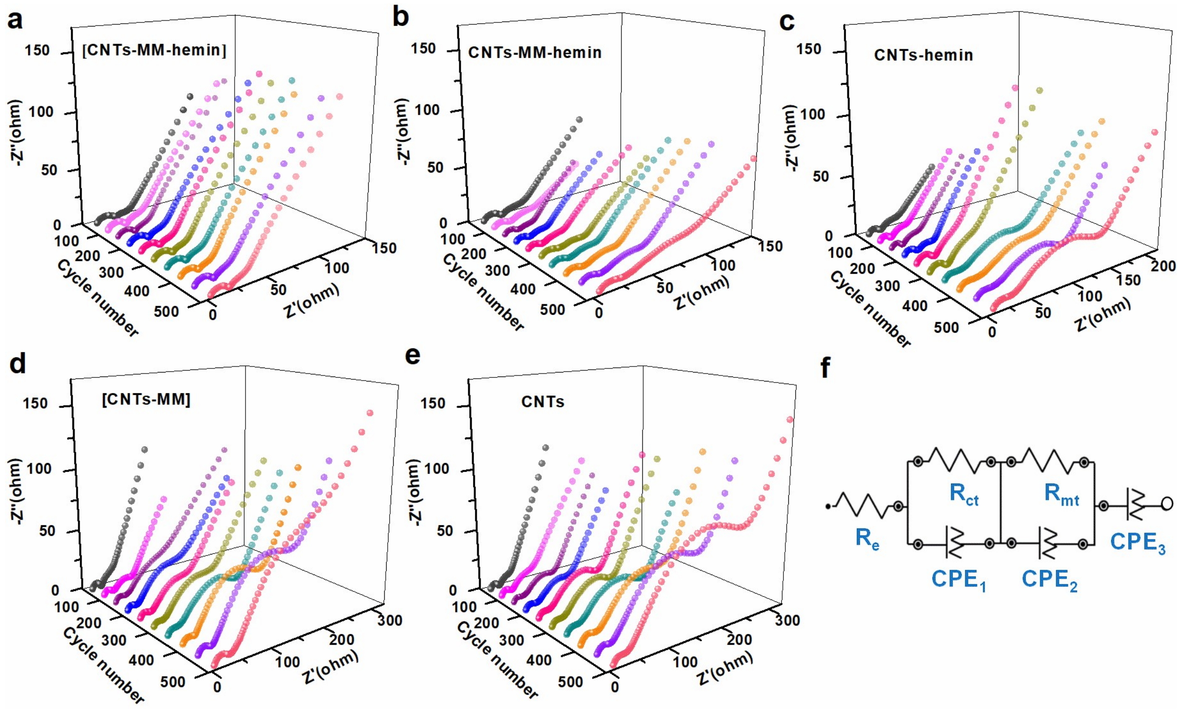

Figure S20. EIS spectra of different cathodes and their equivalent circuit. a–e) The EIS spectra of the cathodes containing [CNTs-MM-hemin] (a), CNTs-MM-hemin (b), CNTs-hemin (c) and [CNTs-MM] (d) and CNT (e) composites after different cycles. f) The equivalent circuit used to fit the obtained EIS spectra in Figure S19a–e (Supporting Information).

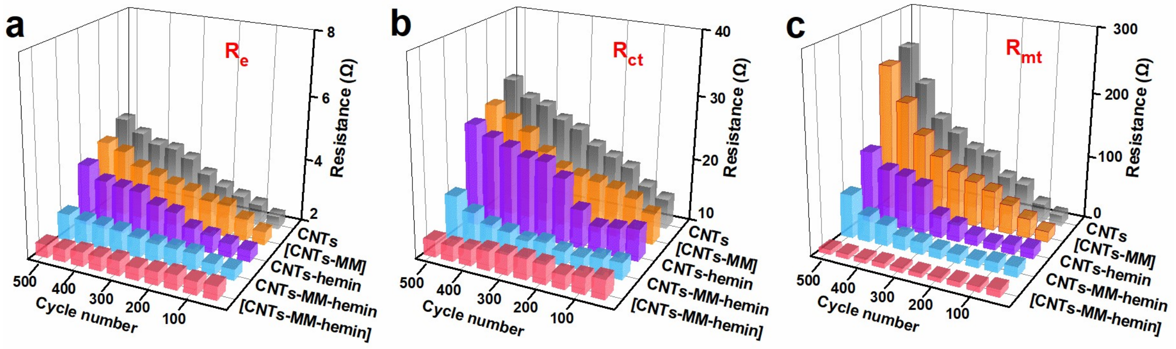

Figure S21. Different resistances from EIS spectra. a–c) The R_e (a), R_{ct} (b) and R_{mt} (c) values of the five cathodes after different cycles.

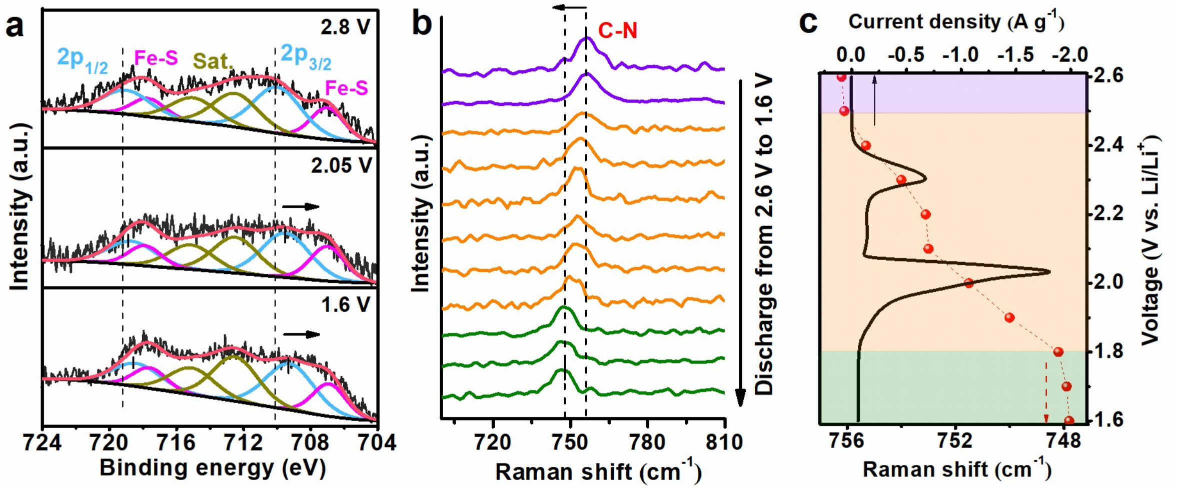

Figure S22. Structural dynamics of [CNTs-MM-hemin] catalysts during discharge. a) Semi-in situ Fe 2p XPS spectra of [CNTs-MM-hemin] cathodes after discharge to specific states. b) In situ Raman spectra of the [CNTs-MM-hemin] cathodes during discharge. c) The discharge electrochemical profile of [CNTs-MM-hemin] cathode in the in situ Raman cell and the potential dependence of Raman shift corresponding to the C–N peak at ≈750 cm^{-1}.

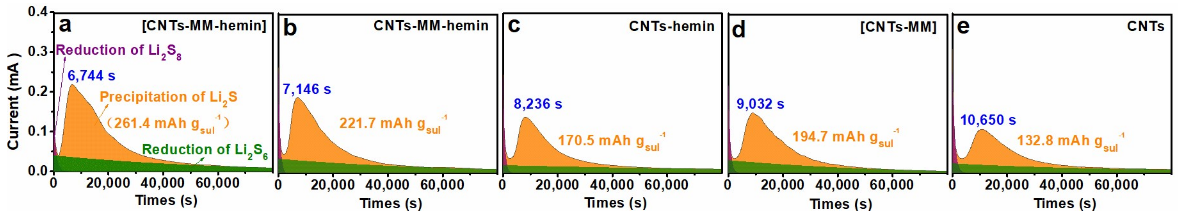

Figure S23. Li_2S nucleation study. a–e) Potentiostatic discharge curves of Li_2S_8/tetraglyme solution at 2.05 V on [CNTs-MM-hemin] (a), CNTs-MM-hemin (b), CNTs-hemin (c), [CNTs-MM] (d) and CNT (e) electrodes, respectively. The purple color indicates the reduction of Li_2S_8, whereas the green color indicates the reduction of Li_2S_6. The precipitation of Li_2S is indicated by orange color.

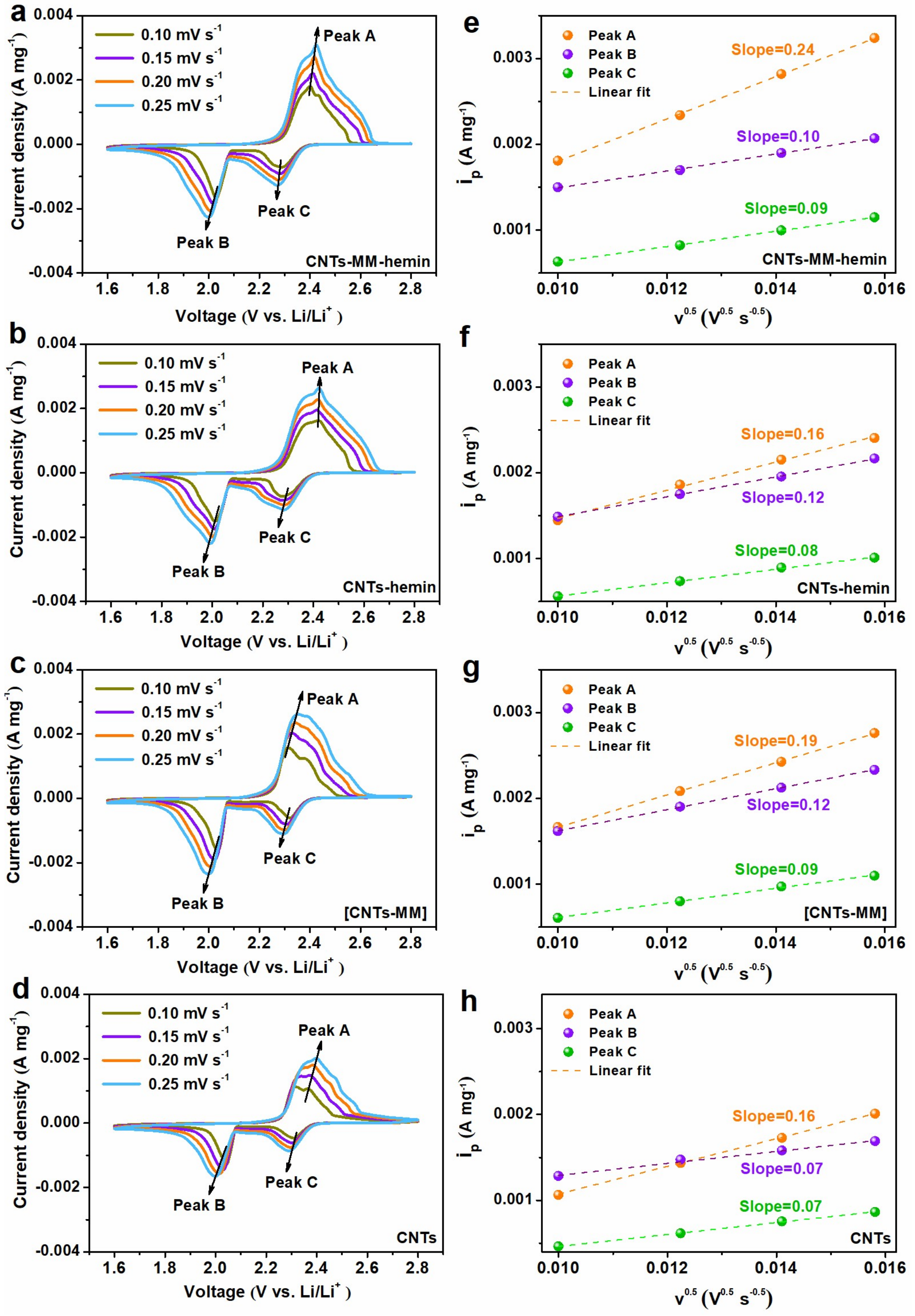

Figure S24. Li^+ diffusion study. a–d) CV curves at different scan rate, and e–h) the corresponding linear fitting based on Equation (1).

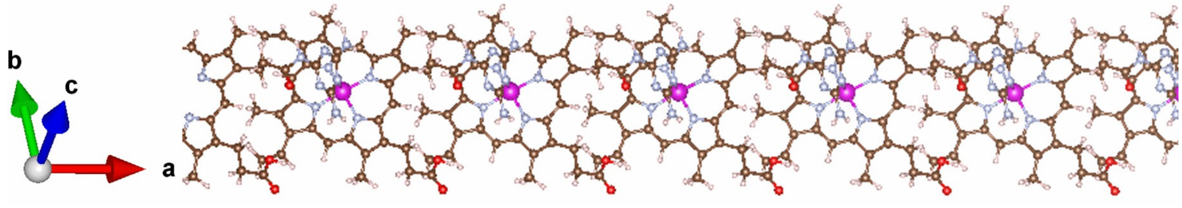

Figure S25. Top view of periodic hemin-MM configuration.

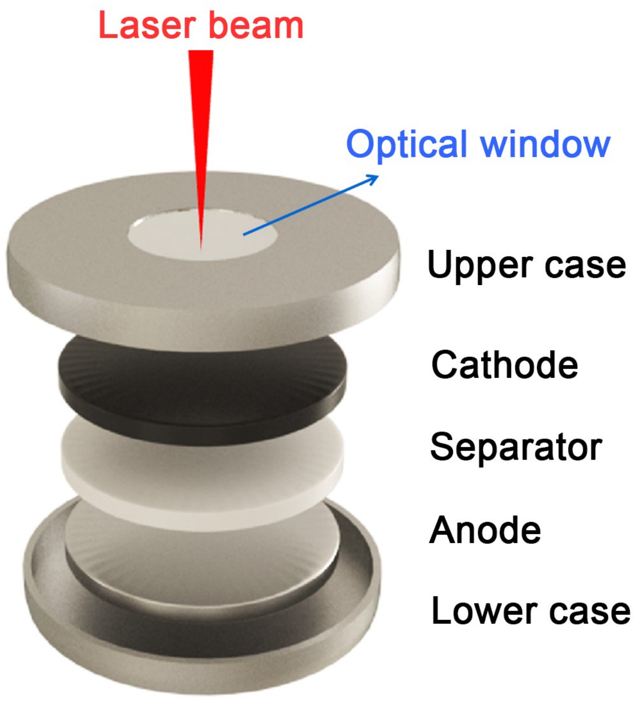

Figure S26. Configuration of the coin cell for in situ Raman experiments.

Table S1. The voltage hysteresis (ΔV, obtained by subtracting the voltage at P_2 from the voltage at P_3, as pointed in Figure S13, Supporting Information) and collection coefficient (I_L/I_H, the ratio of the peak current at 2.05 V (I_L) to the peak current at 2.3 V (I_H)) values derived from the CV profiles in Figure 3a.

**Materials**	**Voltage hysteresis (ΔV) [V]**	**Collection coefficient**
**CNTs**	0.34	2.51
**[CNTs-MM]**	0.31	2.43
**CNTs-hemin**	0.27	2.75
**CNTs-MM-hemin**	0.28	2.68
**[CNTs-MM-hemin]**	**0.24**	**2.86**

Table S2. Characteristics of various materials reported in literatures.

Materials	Sulfur loading [mg cm^{-2}]	Initial performance		Cycling performance			Ref.
		Rate [C]	Capacity [mAh g^{-1}]	Rate [C]	Cycles	Capacity Decay [% per cycle]	
$Fe(OH)_3$@GO	2	0.5	1,569	1	200	0.28	[1]
TS-Ti_3C_2/CNT	1.2	0.2	1,451	1	500	0.09	[2]
$3VO_2$-1VN	1.6–1.8	0.2	1,425	2	800	0.06	[3]
Ti_3C_2	0.7-1	0.2	1,062	2	200	0.25	[4]
NiS@C-HS	1.0	0.2	1,196	0.2	200	0.14	[5]
VS_2	1.0	0.2	1,323	2	100	0.16	[6]
S@KJ-Co_4N	1.1	0.1	1,222	0.5	200	0.09	[7]
MCM-Nb_2O_5	1.5	0.2	1,358	2	500	0.09	[8]
MTN	1.3	0.2	1,315	1	400	0.09	[9]
g-C_3N_4/CNT	1.4	0.1	1,300	1	500	0.03	[10]
Co_3S_4	1.0	0.2	1,330	2	500	0.068	[11]
MoN-NC	1.5	0.2	1,138	1	400	0.08	[12]
MoS_2	1.5	0.2	1,310	0.5	600	0.083	[13]
MHCS@MoS_2	1.5	0.1	1,353	1	500	0.05	[14]
**[CNTs-MM-hemin]**	**1.4**	**0.2**	**1,489**	**1**	**900**	**0.046**	**This work**

Table S3. The D_{Li} values of the five cathodes at different peaks.

D_{Li^+} [cm^2 s^{-1}]	Peak A	Peak B	Peak C
**CNTs**	1.61×10^{-8}	3.08×10^{-9}	3.08×10^{-9}
**CNTs-MM**	2.28×10^{-8}	9.04×10^{-9}	5.08×10^{-9}
**CNTs-hemin**	1.61×10^{-8}	9.04×10^{-9}	4.02×10^{-9}
**CNTs-hemin-MM**	3.61×10^{-8}	6.29×10^{-9}	5.08×10^{-9}
**[CNTs-hemin-MM]**	3.92×10^{-8}	1.61×10^{-8}	7.60×10^{-9}

References

[1] M. A. Al-Tahan, Y. Dong, R. Zhang, Y. Zhang, J. Zhang, *Appl. Surf. Sci.* **2021**, *538*, 148032.

[2] X. Wang, D. Luo, J. Wang, Z. Sun, G. Cui, Y. Chen, T. Wang, L. Zheng, Y. Zhao, L. Shui, G. Zhou, K. Kempa, Y. Zhang, Z. Chen, *Angew. Chem. Int. Ed.* **2020**, *59*, 2.

[3] Y. Song, W. Zhao, L. Kong, L. Zhang, X. Zhu, Y. Shao, F. Ding, Q. Zhang, J. Sun, Z. Liu, *Energy Environ. Sci.* **2018**, *11*, 2620.

[4] Y. Dong, S. Zheng, J. Qin, X. Zhao, H. Shi, X. Wang, J. Chen, Z.-S. Wu, *ACS Nano* **2018**, *12*, 2381.

[5] C. Ye, L. Zhang, C. Guo, D. Li, A. Vasileff, H. Wang, S.-Z. Qiao, *Adv. Funct. Mater.* **2017**, *27*, 1702524.

[6] X. Zhu, W. Zhao, Y. Song, Q. Li, F. Ding, J. Sun, L. Zhang, Z. Liu, *Adv. Energy Mater.* **2018**, *8*, 1800201.

[7] Y. Yu, S. Zhen, S. Cao, P. Wu, G. Ma, A. Li, J. Zhang, *Dalton Trans.* **2021**, *50*, 116.

[8] Y. Tao, Y. Wei, Y. Liu, J. Wang, W. Qiao, L. Ling, D. Long, *Energy Environ. Sci.* **2016**, *9*, 3230.

[9] B. Qi, X. Zhao, S. Wang, K. Chen, Y. Wei, G. Chen, Y. Gao, D. Zhang, Z. Sun, F. Li, *J. Mater. Chem. A* **2018**, *6*, 14359.

[10] X. Wang, G. Li, M. Li, R. Liu, H. Li, T. Li, M. Sun, Y. Deng, M. Feng, Z. Chen, *J. Energy Chem.* **2021**, *53*, 234.

[11]T. Chen, Z. Zhang, B. Cheng, R. Chen, Y. Hu, L. Ma, G. Zhu, J. Liu, Z. Jin, *J. Am. Chem. Soc.* **2017**, *139*, 12710.

[12]P. Wang, N. Li, Z. Zhang, B. Hong, J. Li, K. Zhang, K. Xie, Y. Lai, *J. Mater. Chem. A* **2019**, *7*, 21934.

[13]H. Lin, L. Yang, X. Jiang, G. Li, T. Zhang, Q. Yao, G. W. Zheng, J. Y. Lee, *Energy Environ. Sci.* **2017**, *10*, 1476.

[14]Q. Shao, P. Lu, L. Xu, D. Guo, J. Gao, Z.-S. Wu, J. Chen, *J. Energy Chem.* **2020**, *51*, 262.
